# RGS1 regulates myeloid cell accumulation in atherosclerosis and aortic aneurysm rupture through altered chemokine signalling

**DOI:** 10.1038/ncomms7614

**Published:** 2015-03-18

**Authors:** Jyoti Patel, Eileen McNeill, Gillian Douglas, Ashley B. Hale, Joseph de Bono, Regent Lee, Asif J. Iqbal, Daniel Regan-Komito, Elena Stylianou, David R. Greaves, Keith M. Channon

**Affiliations:** 1 Division of Cardiovascular Medicine, British Heart Foundation Centre of Research Excellence, University of Oxford, John Radcliffe Hospital, Oxford OX3 9DU, UK; 2 Wellcome Trust Centre for Human Genetics, University of Oxford, Oxford OX3 7BN, UK; 3 Sir William Dunn School of Pathology, University of Oxford, Oxford OX1 3RE, UK; 4 Jenner Institute, University of Oxford, Oxford OX3 7DQ, UK

## Abstract

Chemokine signalling drives monocyte recruitment in atherosclerosis and aortic aneurysms. The mechanisms that lead to retention and accumulation of macrophages in the vascular wall remain unclear. Regulator of G-Protein Signalling-1 (RGS1) deactivates G-protein signalling, reducing the response to sustained chemokine stimulation. Here we show that *Rgs1* is upregulated in atherosclerotic plaque and aortic aneurysms. *Rgs1* reduces macrophage chemotaxis and desensitizes chemokine receptor signalling. In early atherosclerotic lesions, *Rgs1* regulates macrophage accumulation and is required for the formation and rupture of Angiotensin II-induced aortic aneurysms, through effects on leukocyte retention. Collectively, these data reveal a role for *Rgs1* in leukocyte trafficking and vascular inflammation and identify *Rgs1*, and inhibition of chemokine receptor signalling as potential therapeutic targets in vascular disease.

## Abstract (web summary)

Vascular inflammation plays a key role in pathogenesis of major vascular diseases. Here the authors show that Regulator of G-Protein Signaling-1 (RGS1) controls macrophage function in the development of vascular inflammation that underlies atherosclerosis and abdominal aortic aneurysms in mice and humans.

## Graphical abstract

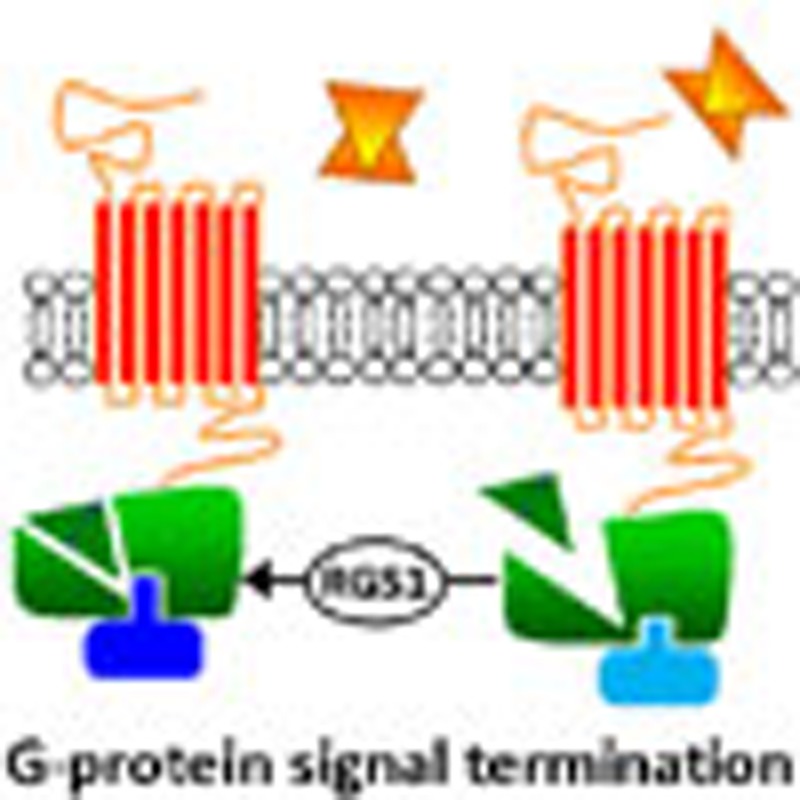

Chemokine signalling plays a key role in leukocyte trafficking in the pathogenesis of vascular inflammation, the underlying cause of cardiovascular diseases such as atherosclerosis and abdominal aortic aneurysms (AAA). Leukocyte activation and chemotaxis is mediated by chemokines binding to multiple G-protein-coupled receptors (GPCRs). Genetic or pharmacological inhibition of chemokines or chemokine receptors results in reduced atherosclerotic plaque formation^1^,^2^,^3^,^4^ and inhibits AAA formation^5^,^6^. Deletion of either *Ccr2* or *Ccr5* decreases plaque formation in mouse models of atherosclerosis that is accompanied by reduced macrophages^1^,^2^, and deletion of *Ccr2* inhibits aortic aneurysm formation^6^,^7^. Although the roles for chemokines and chemokine receptors are well defined in the recruitment of leukocytes to the vascular wall in driving disease progression^8^, it remains less clear how recruited leukocytes accumulate or emigrate in the presence of ongoing chemokine stimulation.

Elucidating these cellular mechanisms would provide strategies targeted at reducing macrophage accumulation or promoting macrophage emigration in the inflamed vasculature. Chemokine receptors couple to Gαi subunits that have an intrinsic GTPase activity, which can be enhanced by RGS proteins, leading to GPCR desensitization. Of the RGS proteins, Regulator of G-Protein Signalling-1 (RGS1) accelerates Gαi GTPase activity and acts to downregulate the response to sustained chemokine activation^9^,^10^. Genome-wide association studies have speculated a link between *Rgs1* and polymorphisms associated with the risk of several chronic inflammatory diseases such as celiac disease, multiple sclerosis and type I diabetes^11^,^12^,^13^. Other studies have identified a role for RGS1 in the control of lymphocyte homeostasis^10^,^14^, but to date no studies have investigated the function of RGS1 in macrophages in vascular inflammation.

Here we report a previously unknown role for RGS1 in monocyte–macrophage trafficking in the development of vascular inflammation. We identified *Rgs1* as a novel candidate gene in atherosclerosis and AAA, and tested the hypothesis that RGS1 is a key modulator of chemokine receptor activity, with critical roles in regulating the vascular inflammatory response by affecting macrophage function. We investigated the effects of *Rgs1* deletion on macrophage recruitment and retention in the artery wall, and elucidate a new requirement for RGS1 in leukocyte accumulation in atherosclerosis and AAA rupture.

## Results

### Vascular inflammation increases *Rgs1* expression

**Figure 1:**
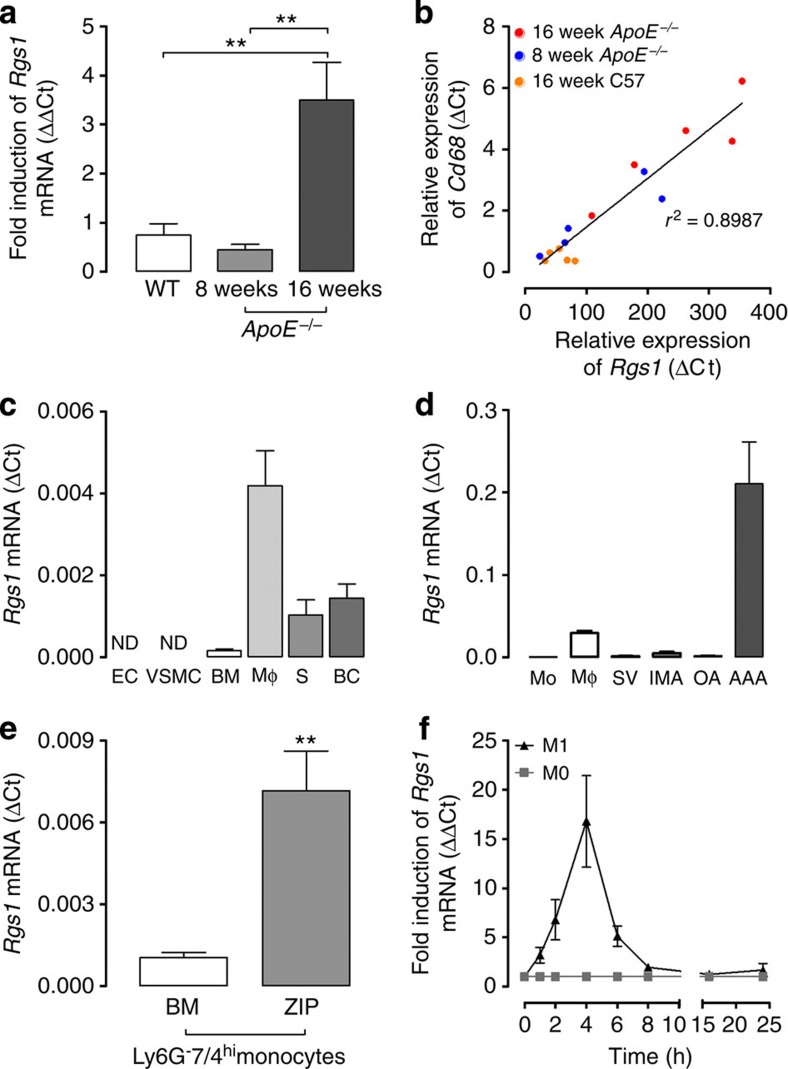
*Rgs1* is upregulated by inflammatory stimuli in activated monocytes. (**a**) Confirmation of *Rgs1* mRNA changes in thoracic aortas of *ApoE*^*−/−*^ mice and wild-type controls on a high-fat diet (*n*=5 per group) by qRT–PCR. (**b**) *Rgs1* expression is correlated with the expression of the macrophage marker *Cd68* in thoracic aortas of *ApoE*^*−/−*^ mice and wild-type controls on a high-fat diet. Each symbol represents an individual mouse (*n*=5 per group) by qRT–PCR. (**c**) qRT–PCR analysis of *Rgs1* mRNA in primary cells isolated from *ApoE*^*−/−*^ mice (*n*=5–6; BC, B cells; BM, bone marrow cells; EC, endothelial cells; MΦ, macrophages; S, splenocytes). (**d**) qRT-PCR analysis of *Rgs1* expression in human tissue and cells. (Mo; Blood monocytes, plaque macrophages from carotid endarterectomies (*n*=3), SV; Saphenous vein and IMA; internal mammary artery from CABGs (*n*=8), OA; omental artery and AAA; abdominal aortic aneurysm from AAA repair patients (*n*=8–11)). (**e**) SV and IMA are from CABGs. qRT–PCR analysis of *Rgs1* mRNA in Ly6G-7/4^hi^ BM monocytes and peritoneal monocytes isolated from zymosan-induced peritonitis (ZIP) in *ApoE*^*−/−*^ mice (*n*=6–7 per group). (**f**) qRT–PCR analysis of *Rgs1* mRNA in bone marrow-derived macrophages from *ApoE*^*−/−*^ mice stimulated with IFN-γ and lipopolysaccharide (M1) and unstimulated (M0) over 24 h presented relative to mRNA in unstimulated cells, set as 1. *P<0.05, **P<0.01 calculated using the Student’s *t*-test (Data in **a** are expressed as mean±s.d. and data in **c**–**f** as mean±s.e.m.).

To identify genes that are specifically regulated with atherosclerosis progression, we used a whole mouse genome array to profile the gene expression in the thoracic aortas from *ApoE*^*−/−*^ mice, comparing older atherosclerotic *ApoE*^*−/−*^ mice with young littermate animals before plaque development (Supplementary Table 1). Among a number of genes already known to play major roles in the development of atherosclerosis such as *Ccl2* (MCP-1), *Rgs1* was identified as one of the novel candidate genes with higher expression in aortas from older *ApoE*^*−/−*^ mice than in aortas from younger *ApoE*^*−/−*^ mice. We confirmed that *Rgs1* mRNA was upregulated in aortas from atherosclerotic *ApoE*^*−/−*^ mice compared with younger *ApoE*^*−/−*^ mice (8 weeks, male mice) or wild-type C57BL/6 mice of the same age (16 weeks, male mice) by quantitative reverse transcriptase-PCR (qRT–PCR) (Fig. 1a). The high expression of *Rgs1* was associated with the high expression of the macrophage marker *Cd68* in individual animals, suggesting that macrophages may be the source of RGS1 in atherosclerotic plaques (Fig. 1b). To test this hypothesis, we quantified *Rgs1* expression in different primary cells isolated from *ApoE*^*−/−*^ mice and found high *Rgs1* mRNA levels in CD68 positive macrophages compared with B cells where *Rgs1* is known to have a non-redundant role (Fig. 1c). In contrast, we did not detect *Rgs1* mRNA in either vascular smooth muscle cells (VSMCs) or endothelial cells, which are also known to be involved in atherosclerotic plaque progression (Fig. 1c).

To extend these findings in the mouse atherosclerosis model to human disease, we first evaluated *Rgs1* expression in macrophages isolated from human carotid artery plaques obtained at endarterectomy. *Rgs1* mRNA levels were detectable in macrophages derived from atherosclerotic plaque (Fig. 1d), compared with blood monocytes. Because of the associations between aortic atherosclerosis, vascular inflammation and the pathogenesis of AAA^15^, we next sought to determine whether *Rgs1* is related to human aneurysms. We analysed the tissue samples of AAA from patients undergoing surgical AAA repair, in comparison with non-diseased control samples of omental artery from the same patients, and to internal mammary artery and saphenous vein samples from patients undergoing coronary artery bypass graft surgery (CABG). We also measured *Rgs1* in CD14-positive blood monocytes from the same AAA patients. *Rgs1* was highly expressed in human AAA tissue compared with non-aneurysmal vascular tissues and blood monocytes (Fig. 1d), suggesting that RGS1 is specifically upregulated in recruited monocytes during vascular inflammation. To test the notion that induction of *Rgs1* might occur during monocyte–macrophage activation and recruitment, we compared *Rgs1* expression during the differentiation of murine M0 to M1 ‘inflammatory’ macrophages, and between bone marrow monocytes and peritoneal monocytes recruited in a model of sterile peritonitis. *Rgs1* was significantly higher in recruited peritoneal monocytes than bone marrow monocytes (Fig. 1e), and upregulated in macrophages during early M1 macrophage differentiation (Fig. 1f). We also confirmed that *Rgs1* was abundant in M1-polarized macrophages differentiated from healthy human peripheral blood mononuclear cells (Supplementary Fig. 1).

### RGS1 reduces macrophage chemotaxis and desensitization

**Figure 2:**
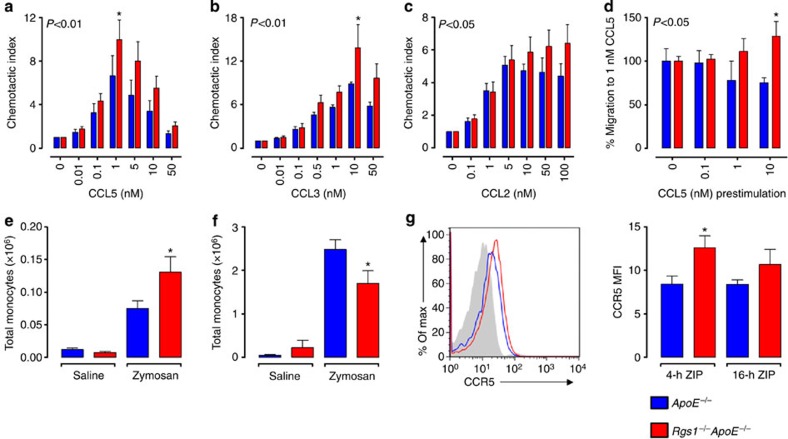
*Rgs1* deletion enhances monocyte–macrophage chemotaxis and impairs homologous desensitization. Migration of peritoneal macrophages from *ApoE*^*−/−*^ and *Rgs1*^*−/−*^ *ApoE*^*−/−*^ mice through an 8-μm filter towards increasing concentrations of recombinant murine (**a**) CCL5, (**b**) CCL3 and (**c**) CCL2 placed in the lower chamber of a Boyden chamber. (**d**) Migration of peritoneal macrophages pretreated with 0, 0.1, 1 and 10 nM CCL5 and exposed to 1 nM CCL5. Quantification of migration is presented relative to results of untreated cells, set as 1. RPMI media was used as a negative control. Graphs indicate migration index±s.e.m. for each treatment group (triplicates; *n*=5–6 per group). *In vivo* chemotaxis was assessed by i.p. injection of 100 μg zymosan and recruited, peritoneal 7/4^hi^Ly6G^−^ monocytes quantified by flow cytometry at (**e**) 4 h and (**f**) 16 h after injection (*n*=2–4 for saline and *n*=6–11 for zymosan) (**g**) The expression of CCR5 on the surface of circulating monocytes after zymosan (*n*=6–7). Mean fluorescence intensity (MFI) is shown for CCR5 on monocytes at 4 h after zymosan above isotype control (grey). *P*<0.01 in **a**,**b**; *P*<0.05 in **c**,**d** calculated by two-way analysis of variance with significance at individual doses indicated by stars calculated by Bonferroni post-tests. *P*<0.05 in **e**–**g** calculated by Student’s *t*-test.

Since *Rgs1* expression in macrophages is high and upregulated with activation, we reasoned that RGS1 would inhibit the migration of macrophages to atherogenic chemokines. We compared the chemotactic responses between *ApoE*^*−/−*^ and *Rgs1*^*−/−*^*ApoE*^*−/−*^ peritoneal macrophages *in vitro*. *Rgs1*^*−/−*^*ApoE*^*−/−*^ macrophage chemotaxis was significantly increased in response to CCL2, CCL3 and CCL5 (Fig. 2a–c) suggesting a broad specificity for RGS1 to Gαi-coupled chemokine receptors. We also confirmed the role of RGS1 in lymphocyte chemotaxis, by showing increased migration of *Rgs1*^*−/−*^*ApoE*^*−/−*^ splenocytes to the homeostatic chemokine CXCL12 (Supplementary Fig. 2) at a similar magnitude to published studies^10^. Because RGS1 promotes the formation of the inactive G-protein heterotrimer and accelerates the termination of chemokine signalling, we tested the effect of RGS1 on chemokine receptor desensitization in macrophages. We observed that RGS1 reduced the migration to sustained CCL5 stimulation of macrophages. Pretreatment of peritoneal macrophages with increasing doses of CCL5 before chemotaxis to 1 nM CCL5 markedly impaired chemotaxis in *ApoE*^*−/−*^ macrophages, whereas *Rgs1*-deficient macrophages continued to migrate, regardless of previous exposure to chemokine (Fig. 2d). To further address the role of RGS1 in myeloid cell chemotaxis, we used a chemokine-dependent model of sterile inflammation—zymosan-induced peritonitis^16^ to assess cellular recruitment *in vivo*. At an early time point after zymosan administration, coinciding with the phase of cellular recruitment to the peritoneum, we observed that the number of monocytes in the peritoneum was significantly increased in *Rgs1*^*−/−*^*ApoE*^*−/−*^ mice (Fig. 2e). However, at 16 h after zymosan administration, when cellular recruitment has plateaued and the resolution phase is beginning, we observed a significant decrease in the numbers of monocytes in *Rgs1*^*−/−*^*ApoE*^*−/−*^ mice (Fig. 2f) suggesting that an early increase in cell number in *Rgs1*^*−/−*^*ApoE*^*−/−*^ mice is then followed by reduced accumulation. To address if there were any alterations in chemokine receptor signalling that may be crucial for trafficking, we assessed CCR5 and CCR2 surface expression on circulating monocytes in mice treated with zymosan. At 4 h, coinciding with the increase in monocytes in the peritoneum of *Rgs1*^*−/−*^*ApoE*^*−/−*^ mice, there was an increase in CCR5 on the circulating monocytes compared with monocytes in *ApoE*^*−/−*^ mice. In contrast, at 16 h, there was no difference in the cell surface level of CCR5 between *Rgs1*^*−/−*^*ApoE*^*−/−*^ and *ApoE*^*−/−*^ monocytes (Fig. 2g). However, CCR2 surface expression was not detectable on circulating monocytes from either *Rgs1*^*−/−*^*ApoE*^*−/−*^ and *ApoE*^*−/−*^ mice after 4 h of zymosan.

### RGS1 modulates macrophage trafficking into the aortic wall

**Figure 3:**
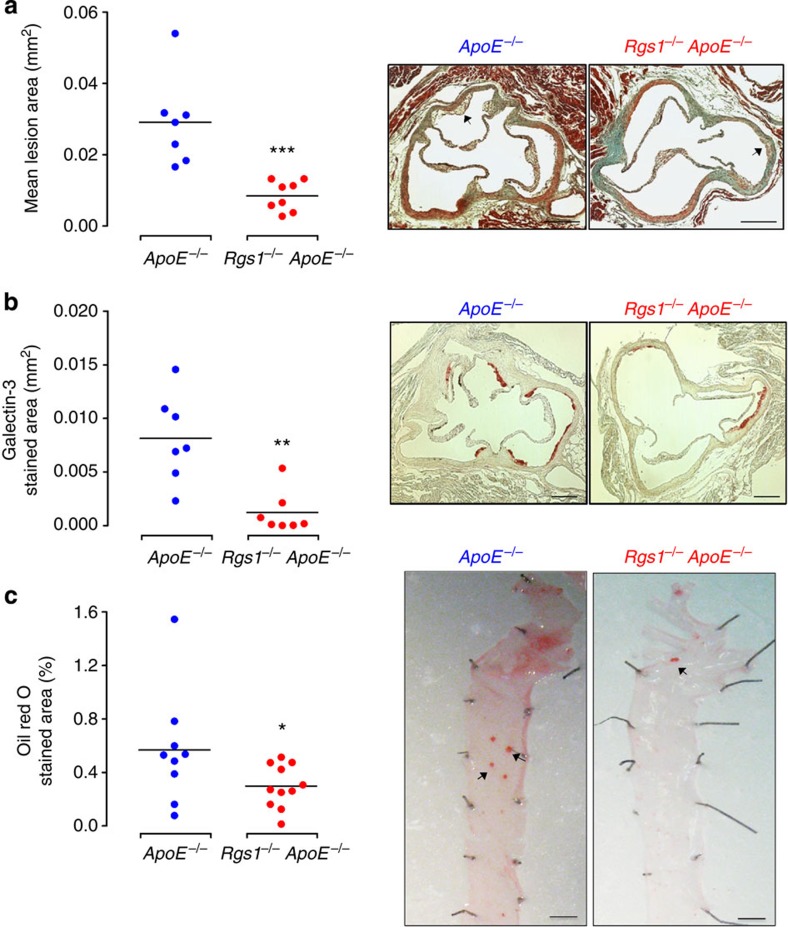
*Rgs1* deficiency reduces atherosclerosis and macrophage content in *ApoE*^*−/−*^mice. (**a**) Atherosclerotic plaque in the aortic roots of 9-week-old mice on a chow diet. Microscopy of massons trichrome stained aortic root lesions. (**b**) Galectin-3-positive macrophage content in the aortic roots of 9-week-old mice on a chow diet. Microscopy of Galectin-3-stained aortic root lesions. (**c**) *En face* atherosclerotic plaque in the aortas of 16-week-old mice on a chow diet. Microscopy of *en face* Oil Red O staining of aortic arches of descending aortas. Each symbol represents an individual mouse (*n*=7–8 per group). Scale bars indicate 0.25 mm for aortic roots and 1 mm for aortas. Arrows indicate atherosclerotic lesions. Values are expressed as mean±s.e.m. ***P<0.001, **P<0.01, *P<0.05 calculated using the Student’s *t*-test.

Since macrophage recruitment is a critical step in atherogenesis, and given that *Rgs1*-deficient macrophages showed an increased migratory response to atherogenic chemokines *in vitro*, we hypothesized that *Rgs1*^*−/−*^*ApoE*^*−/−*^ mice would develop larger atherosclerotic lesions than *ApoE*^*−/−*^ mice as a result of enhanced leukocyte recruitment. To test this hypothesis, we quantified atherosclerotic plaque in *Rgs1*^*−/−*^*ApoE*^*−/−*^ *and ApoE*^*−/−*^ mice at two anatomical sites—the aortic root and the descending aorta. However, in contrast to our hypothesis, the absence of *Rgs1* significantly reduced both atherosclerotic plaque formation in the aortic root of *ApoE*^*−/−*^ mice (Fig. 3a) and reduced plaque macrophage content, quantified by Galectin-3-positive macrophage immunostaining (Fig. 3b) in 9-week-old animals. Similar results were observed by *en face* analysis of the descending aorta, where plaques develop later in 16-week-old animals. *Rgs1*^*−/−*^*ApoE*^*−/−*^ mice had smaller lesions in comparison with *ApoE*^*−/−*^ littermates (Fig. 3c). No differences in lesion size or macrophage content were observed in the aortic root in mice fed a chow diet for 16 weeks or mice on a western-type diet (Supplementary Fig. 3). In addition, no CD3 T-lymphocyte infiltration in the aortic root was observed at any time point (Supplementary Fig. 4). Since Rgs1 has been reported to contribute to T-cell migration^17^, we also characterized Treg cells and antigen-specific cytokine responses of Th1 and Th17 cells in *Rgs1*^*−/−*^*ApoE*^*−/−*^ and *ApoE*^*−/−*^ mice after co-stimulation with anti-CD3/CD28, and found no significant difference between groups (Supplementary Figs 5 and 6). No difference in total serum cholesterol levels or circulating monocyte numbers were found between *Rgs1*^*−/−*^*ApoE*^*−/−*^ and *ApoE*^*−/−*^ mice, indicating that the observed effect was not due to a change in plasma lipids or monocyte numbers, respectively (Supplementary Table 2). In addition, no differences in *in vitro* foam cell formation were observed between *Rgs1*^*−/−*^*ApoE*^*−/−*^ and *ApoE*^*−/−*^ macrophages treated with acLDL over 24 h (Supplementary Fig. 7).

**Figure 4:**
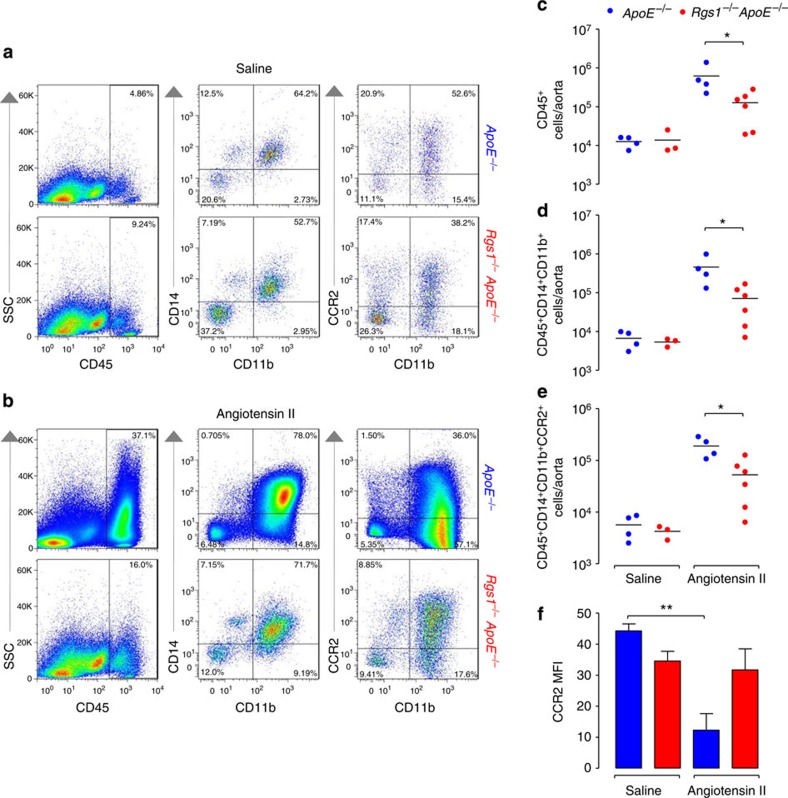
*Rgs1* deficiency reduces aortic inflammatory cell trafficking in Ang II-treated *ApoE*^*−/−*^ mice via CCR2. Flow cytometric analysis of aortic leukocytes in *ApoE*^*−/−*^ and *Rgs1*^*−/−*^ *ApoE*^*−/−*^ mice that received (**a**) saline or (**b**) Ang II infusion at 0.8 mg kg^−1^ per day for 5 days. Representative dot plots shown for gated aortic cells of each positive population from *ApoE*^*−/−*^ and *Rgs1*^*−/−*^ *ApoE*^*−/−*^ mice with representative percentages. Labels on both axes are on a log scale. Quantification of the numbers of (**c**) CD45^+^ cells (**d**) CD45^+^CD14^+^CD11b^+^ cells and (**e**) CD45^+^CD14^+^CD11b^+^CCR2^+^ cells in saline-treated and Ang II-infused mice. Each symbol represents an individual mouse (*n*=3–4 for saline and *n*=4–6 for Ang II). There were two deaths from aneurysm rupture in the *ApoE*^*−/−*^ Ang II group. (**f**) The expression of CCR2 on CD45^+^CD14^+^CD11b^+^ cells in the aorta of saline-treated and Ang II-infused mice (MFI, mean fluorescence intensity). Values are expressed as mean±s.e.m. *P<0.05 and **P<0.01 calculated using the Student’s *t*-test.

To determine the effect of *Rgs1* deficiency on the early influx of myeloid cells into the aortic wall during inflammation, we infused Angiotensin II (Ang II) at 0.8 mg kg^−1^ per day using subcutaneous mini pumps in *ApoE*^*−/−*^ mice, a model known to induce acute aortic leukocyte recruitment and increase blood pressure. Leukocyte content in the thoracic aortas of *Rgs1*^*−/−*^*ApoE*^*−/−*^ and *ApoE*^*−/−*^ mice following Ang II infusion was quantified by enzymatic digestion and flow cytometry (Fig. 4a,b)^18^,^19^. In *Rgs1-*deficient mice, CD45^+^ leukocytes and CD11b^+^ myeloid cells in aortas were reduced more than 10-fold compared with *ApoE*^*−/−*^ mice after 5 days of Ang II infusion (Fig. 4c–e). These cells were CD14+CCR2+, indicative of recruited monocyte–macrophages. Since Ang II mediates monocyte recruitment to the aorta, and AAA formation is largely driven by CCR2 in contrast to CCR5 (refs 6, 18, 19), we assessed CCR2 expression on these cells. We found that CCR2 surface expression on macrophages in the aortas was significantly decreased following Ang II treatment in *ApoE*^*−/−*^ mice (Fig. 4f). However, no reduction in cell surface CCR2 was observed in macrophages from *Rgs1*^*−/−*^*ApoE*^*−/−*^ mice, indicating a lack of receptor desensitization in *Rgs1*^*−/−*^*ApoE*^*−/−*^ mice. Furthermore, between days 3 and 5 of Ang II infusion, two out of six *ApoE*^*−/−*^ mice died from aneurysm rupture, but no deaths occurred in *Rgs1*^*−/−*^*ApoE*^*−/−*^ mice, indicating that *Rgs1* deficiency confers protection from Ang II-induced aortic aneurysm rupture.

**Figure 5:**
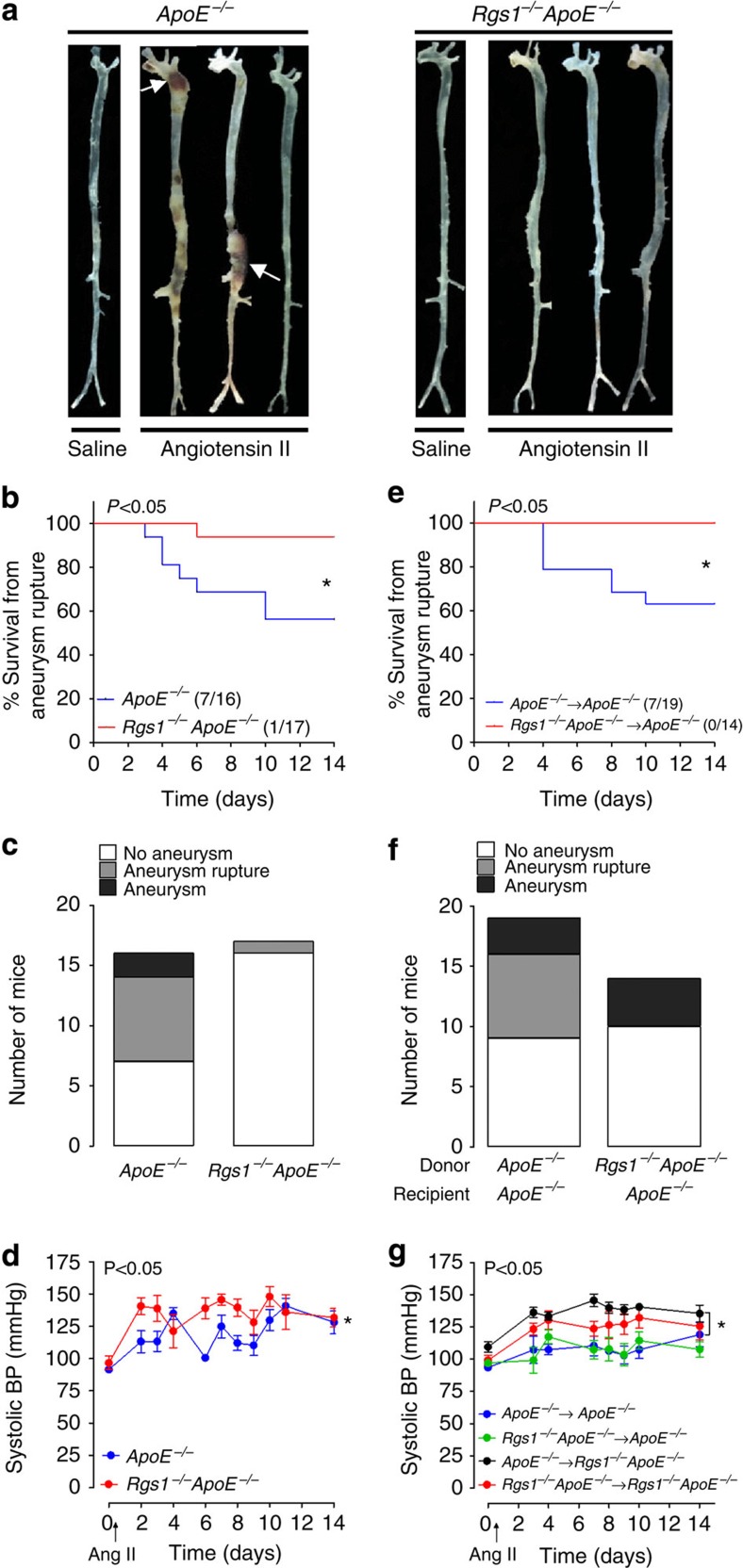
*Rgs1*^*−/−*^ *ApoE*^*−/−*^ mice are protected from Ang II-induced aortic aneurysm rupture. (**a**) *ApoE*^*−/−*^ and *Rgs1*^*−/−*^ *ApoE*^*−/−*^ mice were infused with Ang II or saline for 14 days. Representative photographs showing features of aneurysms induced by Ang II in surviving mice. The arrows indicate typical aneurysms in *ApoE*^*−/−*^ mice. There was no aneurysm formation in the control saline-treated group in both *ApoE*^*−/−*^ and *Rgs1*^*−/−*^ *ApoE*^*−/−*^ mice (*n*=3–4). (**b**) Survival curve of *ApoE*^*−/−*^ and *Rgs1*^*−/−*^ *ApoE*^*−/−*^ mice during Ang II (0.8 mg kg^−1^ per day) infusion. All deaths were due to aortic rupture (**c**) The incidence of Ang II-induced aortic aneurysms in *ApoE*^*−/−*^ mice compared with *Rgs1*^*−/−*^ *ApoE*^*−/−*^ mice. (**d**) The systolic blood pressure (BP) of *ApoE*^*−/−*^ and *Rgs1*^*−/−*^ *ApoE*^*−/−*^ mice that were infused with Ang II (0.8 mg kg^−1^ per day) over 14 days. (**e**) Survival curve of chimeric *ApoE*^*−/−*^ mice during Ang II infusion (3 mg kg^−1^ per day). All deaths were due to aortic rupture. (**f**) The incidence of Ang II-induced aortic aneurysms in chimeric *ApoE*^*−/−*^ mice. (**g**) The systolic blood pressure of chimeric *ApoE*^*−/−*^ and *Rgs1*^*−/−*^ *ApoE*^*−/−*^ mice that were infused with Ang II (0.8 mg kg^−1^ per day) over 14 days. *P<0.05 in **b**,**e** calculated using the *χ*^2^-test (*n*=14–19). *P<0.05 in **d**,**g** calculated using one-way analysis of variance of area under the curve (*n*=5–6). Data in **d**,**g** are expressed as mean±s.e.m.

To further investigate the contribution of RGS1 to Ang II-induced aortic aneurysm formation, we performed 14-day Ang II infusions in *Rgs1*^*−/−*^*ApoE*^*−/−*^ and *ApoE*^*−/−*^ mice, since most aortic ruptures occur within the first 7 days^20^. *ApoE*^*−/−*^ mice were significantly more susceptible to aortic aneurysm rupture in comparison with *Rgs1*^*−/−*^*ApoE*^*−/−*^ mice, with 56% survival in *ApoE*^*−/−*^ mice versus 94% in the *Rgs1*^*−/−*^*ApoE*^*−/−*^group (Fig. 5a,b). We also noted aneurysms at the study end point in surviving *ApoE*^*−/−*^ mice, which were absent in *Rgs1*^*−/−*^*ApoE*^*−/−*^ mice (Fig. 5c). No difference in circulating, bone marrow and spleen monocyte numbers were found between *Rgs1*^*−/−*^*ApoE*^*−/−*^ and *ApoE*^*−/−*^ mice, indicating that the observed effect was not due to a change in monocyte numbers elsewhere (Supplementary Fig. 8). Previous studies have demonstrated that Ang II infusion increases systolic blood pressure in mice^7^. Therefore, to determine whether *Rgs1* deficiency affects Ang II-mediated increases in blood pressure, we measured the systolic blood pressure in both groups. Between days 2 and 10, Ang II treatment increased systolic blood pressure in *Rgs1*^*−/−*^*ApoE*^*−/−*^ mice more than in *ApoE*^*−/−*^ mice (Fig. 5d), demonstrating that protection from aneurysm formation in *Rgs1*^*−/−*^*ApoE*^*−/−*^ mice occurs despite a greater rise in blood pressure and through mechanisms that are independent of Ang II-induced hypertension.

### Leukocyte *Rgs1* deficiency protects against aneurysm rupture

Evidence suggests that chemokines are involved in the modulation of Ang II-accelerated leukocyte recruitment to the vessel wall^21^. It is well known that macrophages are the predominant leukocyte in Ang II ascending aneurysms and AAAs^19^,^22^ and mediate extracellular matrix breakdown that leads to aneurysm formation. We hypothesized that RGS1 may regulate macrophage recruitment and retention via chemokine receptor desensitization and limit macrophage egress and thus augment Ang II-induced AAA development and rupture.

To test this hypothesis, we performed *Rgs1*^*−/−*^*ApoE*^*−/−*^ and *ApoE*^*−/−*^ bone marrow transplantation into irradiated *ApoE*^*−/−*^ mice, to generate bone marrow chimeric animals. Four weeks after transplantation, we tested for *Rgs1* and *ApoE* DNA by PCR in blood from irradiated mice, to confirm complete engraftment. At 6 weeks after engraftment, mice were infused with Ang II for 14 days at 3 mg kg^−1^ per day. We chose this dose as Ang II at 0.8 mg kg^−1^ per day was not sufficient to induce aortic aneurysms in irradiated *ApoE*^*−/−*^ mice in comparison with non-irradiated mice receiving this dose, despite being sufficient to induce hypertension (Fig. 5g).

We observed that recipient mice transplanted with *ApoE*^*−/−*^ bone marrow were more prone to aortic aneurysm rupture in comparison with mice receiving *Rgs1*^*−/−*^*ApoE*^*−/−*^ bone marrow (Fig. 5e,f). Over 14 days of Ang II infusion, we found similar survival of *ApoE*^*−/−*^ recipient mice with *ApoE*^*−/−*^ donor marrow when compared with that in non-chimeric *ApoE*^*−/−*^ mice from our original study. In contrast, *ApoE*^*−/−*^ recipient mice receiving *Rgs1*^*−/−*^*ApoE*^*−/−*^ donor marrow were protected from Ang II-induced AAA formation. Consistent with our earlier findings, there was a significant difference in systolic blood pressure between *ApoE*^*−/−*^ mice transplanted with *ApoE*^*−/−*^ bone marrow and *Rgs1*^*−/−*^*ApoE*^*−/−*^ mice transplanted with *ApoE*^*−/−*^ bone marrow (Fig. 5g), revealing a role for vascular wall RGS1 in the control of Ang II-induced blood pressure. Taken together, these results suggest that *Rgs1* expression in bone marrow-derived cells, rather than vascular cells, is crucial for aortic aneurysm rupture.

### RGS1 promotes leukocyte accumulation in aneurysms

**Figure 6:**
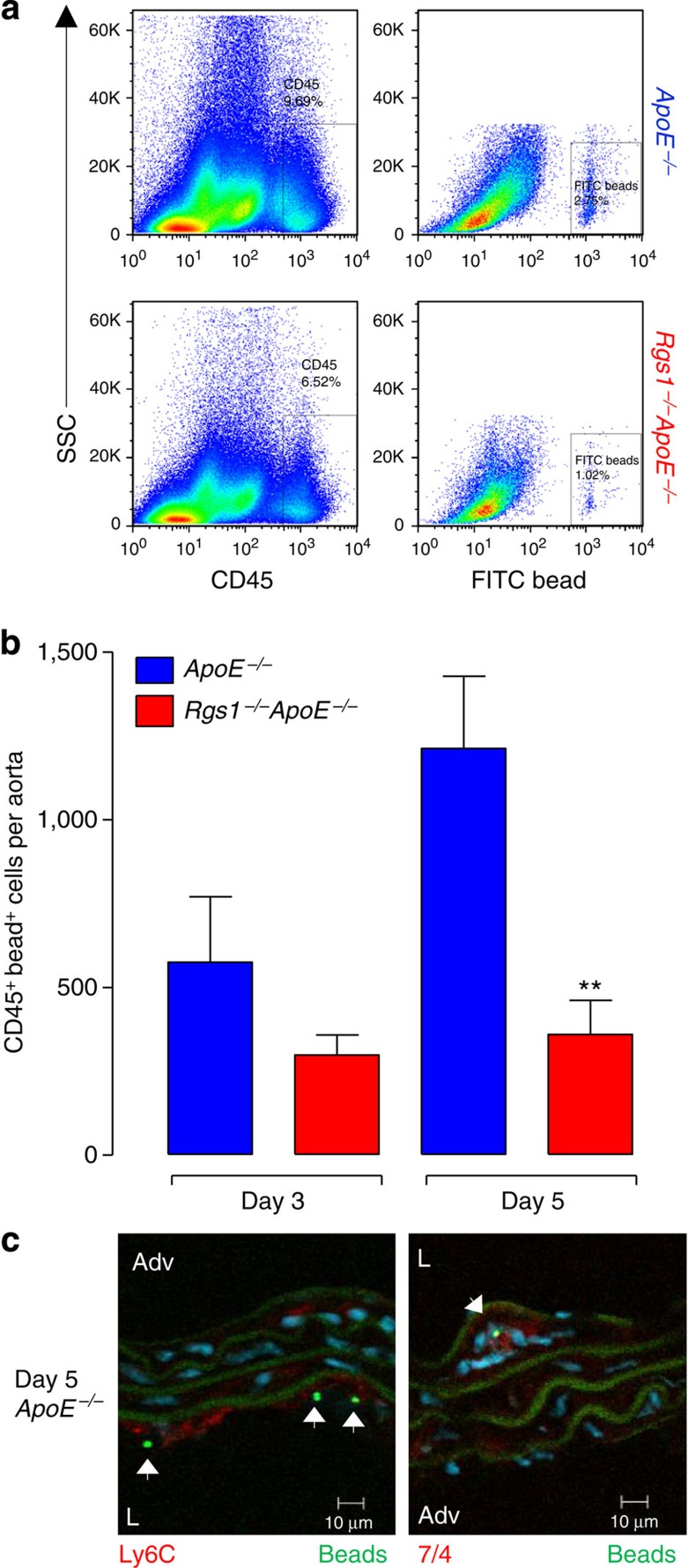
RGS1 promotes leukocyte accumulation in the aortic wall during Ang II-induced vascular inflammation. Flow cytometric analysis of bead-labelled aortic leukocytes in *ApoE*^*−/−*^ and *Rgs1*^*−/−*^ *ApoE*^*−/−*^ mice that received Ang II infusion at 0.8 mg kg^−1^ per day for 5 days following fluorescent bead labelling of circulating inflammatory monocytes. (**a**) Representative dot plots shown for gated aortic cells of each positive population from *ApoE*^*−/−*^ and *Rgs1*^*−/−*^ *ApoE*^*−/−*^ mice with representative percentages. Labels on both axes are on a log scale. (**b**) Quantification of the number of bead-labelled CD45^+^ cells in aortas of Ang II-infused mice at days 3 and 5. (**c**) Immunofluorescence microscopy of abdominal aortas from *ApoE*^*−/−*^ mice at day 5 after Ang II infusion and bead labelling stained for Ly6C and 7/4 (red), 4′,6-diamidino-2-phenylindole (blue). Arrows indicate the presence of cells containing fluorescent beads (green) on the luminal side (L) of the aorta or within the internal elastic laminar (green autofluorescence). Adv, adventitia. **P<0.01 calculated using the Student’s *t*-test (*n*=5–7 per group. Data in **b** are expressed as mean±s.e.m.).

To specifically address the role of *Rgs1* in the accumulation or emigration of monocyte-derived cells in the aortic wall during aneurysm development, we used a pulse-chase approach to track bead-labelled monocytes in aortas following Ang II infusion. Inflammatory 7/4^hi^ monocytes were labelled *in vivo* with fluorescent latex microbeads and administered intravenously (i.v.) at the time of osmotic mini pump implantation^23^,^24^. Aortic cell numbers were quantified by flow cytometry at days 3 and 5 after bead injection (Fig. 6a). At 3 days post Ang II infusion, the time of peak monocyte recruitment from the bloodstream, bead-positive leukocyte content in aortas was similar between *ApoE*^*−/−*^ and *Rgs1*^*−/−*^*ApoE*^*−/−*^ mice, implying that monocyte recruitment was similar between the groups (Fig. 6b). However, by day 5 after the initiation of Ang II infusion, bead-positive CD45^+^ cells were significantly higher in *ApoE*^*−/−*^ aortas compared with *Rgs1*^*−/−*^*ApoE*^*−/−*^ mice, suggesting that RGS1 promotes the accumulation of *ApoE*^*−/−*^ monocytes in aortic tissue, rather than emigration. Importantly, these findings likely underestimate the magnitude of accumulation of bead-labelled monocytes, since we found several *ApoE*^*−/−*^ mice with aneurysms at the time of harvest, which were excluded from the analysis because of the confounding effect of blood cells trapped in aneurysms, to the flow cytometric analysis of aortic cells. Bead-labelled monocytes localized to areas of the subintimal space of the vessel wall and were 7/4 and Ly6C positive (Fig. 6c). In contrast to the difference in bead-labelled cells in the aortic wall, there was no difference in the number of circulating bead-labelled inflammatory monocytes between *ApoE*^*−/−*^ and *Rgs1*^*−/−*^*ApoE*^*−/−*^ mice at day 3 or 5 (Supplementary Fig. 9). Together these data suggest that RGS1 is a mediator of inflammatory monocyte accumulation in aortic aneurysms.

## Discussion

Leukocyte recruitment and accumulation leading to vascular inflammation are critical components of major vascular diseases such as atherosclerosis and AAA. Thus, the signals that regulate these processes are crucial for understanding the underlying causes of inflammatory diseases and to identifying novel therapies. Chemokines and their receptors are rational therapeutic targets in vascular inflammation as indicated by several gene targeting^6^,^25^ and inhibition studies^4^,^5^. However, functional redundancy within chemokines and chemokine receptors confers limited potential as therapeutic targets. Targeting downstream pathways that modulate chemokine receptor signalling is an alternative strategy but the mechanisms behind this regulation are not fully elucidated.

**Figure 7:**
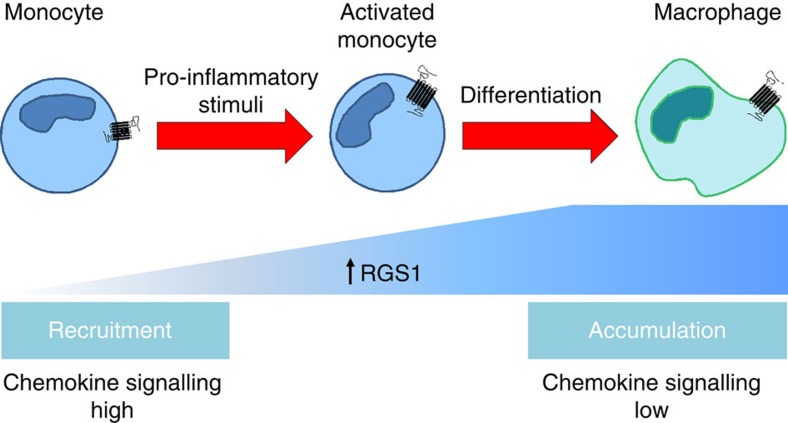
Schematic outline of the regulation of RGS1 in monocyte–macrophages. The expression of *Rgs1* in non-activated circulating monocytes is low and upregulated with monocyte activation by pro-inflammatory stimuli during the recruitment phase. Monocytes differentiate into inflammatory macrophages, whereupon they increase their expression of *Rgs1*, when they are required to generate an inflammatory response. RGS1 terminates chemokine signalling and thereby reduces the capacity for cell migration, which results in the accumulation of macrophages in the subintimal space. RGS1 might be overactivated in the local environment and perpetuate inflammation in the vessel wall. Thus, the differential expression of *Rgs1* in monocytes and macrophages may affect early atherosclerotic lesion and aortic aneurysm progression *in vivo*. This may also be applicable in other inflammatory diseases where dysregulation of monocyte–macrophage trafficking can influence the result of an inflammatory response.

For the first time, we provide new evidence for a role for RGS1, a downstream mediator of GPCR signalling, in the recruitment and accumulation of leukocytes to the aorta during vascular inflammation in atherosclerosis, aortic aneurysm formation and aneurysm rupture. The major findings of this study are, first, that *Rgs1* is upregulated in atherosclerotic vessels and in AAA, is low in circulating monocytes but is greatly upregulated in response to activation and macrophage differentiation (Fig. 7); second, that *Rgs1* deficiency increases macrophage chemotaxis and reduces chemokine receptor homologous desensitization; and third, *Rgs1* deficiency protects against early atherosclerotic plaque and aortic aneurysm rupture in *ApoE*^*−/−*^ mice, due to reduced accumulation of leukocytes in the artery wall. Thus, RGS1 contributes to the persistence of macrophages in the initial stages of atherosclerosis and promotes aortic aneurysm formation and rupture.

Our identification of *Rgs1* in a gene expression analysis in atherosclerotic vessels from older *ApoE*^*−/−*^ mice compared with younger *ApoE*^*−/−*^ mice, and in macrophages from human plaques, is in keeping with other studies in human atherosclerotic arteries. Such studies have reported *Rgs1* upregulation in advanced calcified aortic valve stenosis^26^, atherosclerotic coronary arteries^27^ and unstable carotid artery plaques^28^. Plaque instability can lead to rupture, the underlying cause of myocardial infarction. Our study now reveals that *Rgs1* is specifically upregulated in monocytes and macrophages by inflammatory stimuli, indicating that *Rgs1* expression is high in the recruited cells that contribute to plaque formation and/or is further upregulated in the cells within the fatty-streak lesions or aneurysms of *ApoE*^*−/−*^ mice, and in human aneurysms. The more complex plaque lesions characteristic of more advanced atherosclerosis are influenced by multiple processes such as cholesterol cleft formation, VSMC proliferation, cellular apoptosis and non-cellular lipid accumulation, rather than the more dominant effect of monocyte recruitment and macrophage accumulation that dominate early lesion formation. Our finding supports the notion that RGS1 expression acts to reduce ongoing chemokine signalling in recruited cells, leading to accumulation at sites of inflammation, such that in *Rgs1*^*−/−*^*ApoE*^*−/−*^ mice, inflammatory cell accumulation is significantly reduced.

The functional role of RGS1 has previously been limited to lymphocytes and the control of lymphocyte migration to lymphoid homing chemokines. Several studies have suggested both atheroprotective^29^,^30^,^31^ and proatherogenic^32^,^33^,^34^ roles for the different B-cell and T-cell subsets. Hypercholesterolemic *Rag1*^*−/−*^ mice have reduced atherosclerosis at 16 weeks on a chow diet, but no differences on a western-type diet^35^. The functional roles of lymphocytes in AAA are less clear. Both B cells and T cells have been detected within AAA^36^ but are thought to have minor roles given that *Rag1*^*−/−*^ mice show modest protection from AAA formation^37^. *Rgs1*^*−/−*^ B and T cells show increased migration to CXCL12 (refs 10, 14) and *Rgs1*^*−/−*^ B cells still retain this exaggerated response after pre-exposure due to impaired desensitization^9^,^10^. In addition, when *Rgs1*^*−/−*^ and wild-type T cells are transferred in the inflammatory colitis model in *Rag2*-deficient mice, wild-type mice were more susceptible to colitis, presumably through RGS1 repressing T-cell egress from the gut^14^ associating RGS1 in having a key role in leukocyte accumulation. We examined both the T-cell and B-cell phenotype in of *Rgs1*^*−/−*^*ApoE*^*−/−*^ mice and found no significant alterations, suggesting RGS1 in lymphocytes in the context of atherosclerosis and AAA has a less significant role to that in myeloid cells in vascular inflammation.

Although the findings of this study implicate RGS1 in the formation of fatty-streak lesions and aortic aneurysm rupture, identification of the downstream signalling pathways by which macrophages accumulate remains to be determined. Monocyte–macrophage trafficking to and from the inflamed vasculature is regulated by a number of different mechanisms, such as adhesion, differentiation, retention, proliferation, apoptosis and egress which are also regulated by chemokines. We observed that RGS1 deletion increased macrophage migration to CCL2 and CCL5. Both of these chemokines are involved in monocyte recruitment to atherosclerotic lesions^1^,^23^,^25^ and their respective receptors are high on inflammatory Ly6C^hi^ monocytes^23^, which were the cells recruited in our bead-tracking studies in aortic aneurysms, suggesting RGS1 acts as a ‘stop’ signal downstream of these receptors to reduce signalling and migration. A minor role for RGS1 in reducing integrin-dependent adhesion through the *N*-formyl-methionine-leucine-phenylalanine receptor has been reported in a transfected B-cell line L1.2, although the response was less sensitive to RGS1 action on chemotaxis^38^. Atherosclerotic plaque regression and pro-resolution pathways are expected to act via inhibiting macrophage accumulation, by promoting macrophage egression or by recruiting patrolling Ly6C^lo^ monocytes in reparative processes. Studies in models of atherosclerosis regression suggest that macrophages exhibit a dendritic cell-like state and emigrate from lesions to lymph nodes in a CCR7-dependent manner^39^. RGS1 has been reported to regulate CCR7-mediated T-cell chemotaxis to CCL19 (ref. 14), a chemokine receptor pairing not explored in this study, but a process that could bring about resolution of inflammation. Neuronal guidance molecules such as Netrin-1 and Semaphorin 3E inhibit macrophage chemotaxis and promote the persistence of inflammation by retaining macrophages in the plaque^40^,^41^. These molecules like RGS1, have immunomodulatory functions in the migration and activation of macrophages, indicating the significance of negative regulators in chronic inflammatory diseases.

Our study identifies therapeutic targeting of RGS1 to reduce local vascular inflammation as a new rational strategy for the treatment of cardiovascular diseases. RGS proteins have previously been identified as drug targets, but further understanding on their regulation is needed^42^,^43^. Current strategies have focused on altering RGS protein interactions with Gα protein subunits or the localization or expression of a particular RGS protein in a defined cell^44^. The structure of RGS1 and its binding sites to Gαi protein is known^45^ and so it is logical to propose that an agent to prevent RGS1 binding to its Gαi protein in macrophages, in chronic inflammation may have therapeutic potential. In summary, we have demonstrated a new requirement for RGS1 as a key regulator of chemokine receptor signalling and leukocyte trafficking in vascular inflammation. This provides new insights into the mechanisms and importance underlying the recruitment and retention of leukocytes in vascular inflammation and presents new strategies in targeting RGS1 in limiting atherogenesis and AAA formation.

## Methods

### Mice

The generation of *Rgs1*^*−/−*^ mice has been previously described^10^. *Rgs1*^*−/−*^ mice were crossed onto an *ApoE*^*−/−*^ background (Charles River, UK) to generate matched litters of *Rgs1*^*−/−*^*ApoE*^*−/−*^ and *ApoE*^*−/−*^ mice. Mice were housed in individually ventilated cages with 12-h light/dark cycle and controlled temperature (20–22 °C). Standard chow (B & K Universal Ltd, UK) and water were available *ad libitum*. All animal studies were conducted with ethical approval from the Local Ethical Review Committee and in accordance with the UK Home Office Animals (Scientific Procedures) Act 1986.

### Human blood and tissue sampling

Subjects undergoing open AAA repair were prospectively recruited from the Oxford Abdominal Aortic Aneurysm study. Baseline characteristics of each participant were recorded. For each subject, peripheral venous blood was collected after overnight fasting, before commencement of the surgery. During the surgery, a wedge of abdominal omentum containing a segment of omental artery was identified and biopsied *en bloc*. Isolation of omental artery was performed immediately in the operating theatre. The omental artery segment was cleared of perivascular tissue and snapped frozen. Before incision of the aortic aneurysm, a marker pen was used to denote the cross-section of maximal dilatation according to visual inspection. A longitudinal strip of the aneurysm wall along the incision was then excised. The aneurysm tissue was stripped off perivascular tissue and mural thrombus. The tissue at the maximal dilatation was isolated, divided into smaller segments and snap frozen for subsequent analysis. The study was approved by the Oxford regional ethics committee (Ethics Reference: 13/SC/0250). All subjects gave written informed consent before the study procedure. Human carotid artery plaque macrophages from endarterectomy specimens were isolated following enzymatic digestion of intimal artery segments and *ex vivo* culture^46^. Human saphenous vein and internal mammary artery samples from coronary artery bypass graft surgery patients were dissected, excess adventitia removed and the lumen flushed gently using an insulin syringe to remove blood, before snap freezing for RNA^47^.

### Gene expression profiling

Thoracic aortas from either 8- or 16-week-old *ApoE*^*−/−*^ mice fed a high-fat diet were homogenized in TRIzol reagent (Sigma-Aldrich, UK) and total RNA isolated using RNeasy kits (Qiagen, UK) and reverse transcribed to complementary DNA (cDNA) using SuperScript II Reverse Transcriptase (Invitrogen, UK). Gene expression was measured using a custom-built whole mouse gene array (12,000 genes) with 70mer probes, analysed using the Lucidea system using the Wellcome Trust Centre for Human Genetics Core Genomics Facility. Each sample was arrayed across six slides with two-colour analysis, with reference samples and included a dye-swap. Data analysis was performed using GeneSpring software. The microarray data have been deposited in the NCBI Gene Expression Omnibus under the accession code GSE65494.

### Quantitative real-time RT–PCR

Total RNA was isolated using RNeasy kits (Qiagen) and reverse transcribed to cDNA using SuperScript II Reverse Transcriptase (Invitrogen). Quantitative real-time PCR was performed with 10–50 ng of cDNA on an iCycler IQ real-time detection system (Bio-Rad Laboratories, UK). Gene expression was determined using TaqMan Gene Expression Assays (Applied Biosystems, UK; Supplementary Table 3) relative to the level of the house keeping genes β-actin for mouse, and GAPDH for human using real-time RT–PCR. Relative quantitation of gene expression was performed using the comparative Ct method (ΔΔCt).

### Primary cell isolation and culture

Primary cells and whole thoracic aortas were obtained from 8–16-week-old *ApoE*^*−/−*^ and *Rgs1*^*−/−*^*ApoE*^*−/−*^ mice. Mouse peritoneal macrophages were isolated by peritoneal lavage 4 days after an intraperitoneal (i.p.) injection of 2% BioGel polyacrylamide beads (Bio-Rad Laboratories). Cells were washed in PBS and used for chemotaxis assays or adhered for 2 h in OptiMEM (0.2% bovine serum albumin, BSA) before washing and cell lysis. Mouse splenocytes were isolated by passing spleen pieces through a 70-μm cell strainer. The cell suspension was resuspended in hypotonic lysis buffer (one part Tris-HCl 0.17 M, pH 7.2 to nine parts NH_4_Cl) to lyse red blood cells and washed before lysis for RNA. Purification of B cells was performed by magnetic cell sorting (Miltenyi Biotec, UK) of splenocytes and confirmed by flow cytometry. B cells (>90% pure) were isolated by positive selection using anti-B220 microbeads (Miltenyi Biotec). Bone marrow cells were isolated by flushing the femur and tibia with PBS and a single-cell suspension prepared by passing through a 70-μm cell strainer before lysis. For bone marrow-derived macrophages, bone marrow cells were isolated and the cell suspension plated into Petri dishes with DMEM-F12 (supplemented with 1% penicillin–streptomycin, 1% L-glutamine, 10% fetal bovine serum and 15% L929-cell-conditioned media). Cells were cultured for 7 days in a humidified atmosphere at 37 °C with 5% CO_2_ and then harvested and polarized to M1 inflammatory macrophages with lipopolysaccharide (100 ng ml^−1^; Sigma-Aldrich) and interferon-γ (20 ng ml^−1^; Peprotech EC) for 24 h, compared with untreated (M0).

Primary endothelial cells were isolated from PBS-perfused lung tissue. Lungs were finely minced and digested in an enzyme solution of DMEM containing 0.18 U ml^−1^ Liberase Blendzyme 3 (130 μl; Roche, UK) and 0.1 mg ml^−1^ DNase I (100 μl; Roche) for 1 h at 37 °C with gentle agitation. Purification of endothelial cells was performed by magnetic cell sorting (Miltenyi Biotec) by positive selection using anti-CD31 microbeads (Miltenyi Biotec). Primary VSMCs were isolated by the aortic explant method. Aortas were cut into small segments following endothelial cell denudation and adhered onto 2% gelatin-coated wells and cultured in DMEM complete growth media for 2 weeks. Outgrowing VSMCs from explants of aortic tissue were harvested using Trypsin/EDTA and pelleted for RNA. Mouse peritoneal monocytes were isolated by peritoneal lavage 24 h after i.p. injection of 10 μg Zymosan A (Sigma-Aldrich) and then purified by magnetic cell sorting (Miltenyi Biotec) and confirmed by flow cytometry. Monocytes (>90% pure) were isolated by positive selection using anti-7/4 microbeads (Miltenyi Biotec). Peripheral blood human mononuclear cells were isolated by density-gradient centrifugation (MP Biochemicals, UK). Purification of monocytes was performed by magnetic cell sorting (Miltenyi Biotec) and confirmed by flow cytometry. Monocytes (>90% pure) were isolated by positive selection using anti-CD14 microbeads (Miltenyi Biotec).

### Chemotaxis assays

Cells were resuspended in RPMI (25 mM HEPES and 0.1% BSA) to a cell density of 5 × 10^6^ cells ml^−1^. Eighty μl of the cell suspension was placed on top of 96-well Neuroprobe ChemoTx membranes (5.7 mm diameter, 8 μm pore size; Receptor Technologies, UK) and allowed to migrate towards recombinant murine CCL2, CCL3 or CCL5 (Peprotech EC) or RPMI in lower chambers (320 μl per well) for 4 h at 37 °C, 5% CO_2_. To assess desensitization, macrophages were prestimulated with 0, 0.1, 1 or 10 nM CCL5 for 10 min followed by washing to remove chemokine and then allowed to migrate towards 1 nM CCL5. Cell migration was quantified from fluorescent microscopic images of cells on the underside of the membranes, with a minimum of three replicate wells per treatment.

### Zymosan-induced peritonitis

Mice were injected i.p. with 100 μg of zymosan A (Sigma-Aldrich) diluted in 0.5 ml of PBS or PBS alone. Four or 16 h later, the mice were killed and the peritoneal cavity was lavaged with 5 ml of PBS+2 mM EDTA. Peritoneal exudate cells were stained with antibodies against Ly6G (BD Biosciences) and 7/4 (AbD Serotec, UK) and analysed by flow cytometry to assess monocyte recruitment.

### Atherosclerosis analysis

Mice were fed a normal mouse chow (B & K Universal, UK) and harvested at 9 or 16 weeks of age. For the high-fat diet cohort, 8-week-old mice were fed a western-type diet for 8 weeks and harvested at 16 weeks of age. Atherosclerotic lesion size was assessed in paraffin-embedded aortic root sections stained with Masson-Goldner trichrome (Merck, Germany). The average lesion size was calculated from three sections taken at 100-μm intervals starting from the section showing all three aortic cusps. The infiltration of macrophages into aortic lesions was analysed using anti-Galectin-3 (BD Pharmingen, UK) immunostaining (Supplementary Table 4). Aortic lipid deposition was assessed in fixed aortas stained with Oil red O (Sigma-Aldrich) from mice fed a chow diet for 16 weeks. Aortic roots were visualized and imaged (coolSNAP-pro camera, Roper Scientific, Leica DMRBE microscope and the lesion area and Galectin-3-positive areas were quantified from digitized microscopic images using Image-Pro Plus (Media Cybernetics, USA).

### Ang II infusion and blood pressure recordings

Eight- to 16-week-old male mice were anaesthetized with isoflurane by inhalation and osmotic mini pumps (Alza Corp, USA) delivering saline or Ang II (0.8 or 3 mg kg^−1^ per day; Sigma-Aldrich) for 3, 5 or 14 days were implanted subcutaneously. Systolic blood pressure was measured using a non-invasive computerized tail-cuff system in 16-week-old conscious mice following a 1 week training period (Visitech BP2000, Visitech Systems Inc., USA).

### Flow cytometry

Descending aortas from the aortic arch to femoral bifurcations were microdissected and digested in an enzyme solution containing 60 U ml^−1^ DNase I, 60 U ml^−1^ Hyalronidase, 450 U ml^−1^ Collagenase I and 125 U ml^−1^ Collagenase XI (all enzymes from Sigma-Aldrich) at 37 °C (ref. 18). A single-cell suspension was prepared by passing aortic pieces through a strainer for subsequent flow cytometry staining. Isolated aortic cells were antibody stained for the surface markers PE-Cy7-conjugated CD45 (BD Pharmingen), total PerCP-conjugated CD11b (BD Pharmingen), PE-conjugated CD14 (eBioscience, UK), APC-conjugated CCR2 (R&D Systems, UK) with appropriate isotype controls^19^ (Supplementary Table 5). Absolute cell counts were performed by ratio to a known quantity of calibration beads added to each sample (CaliBrite, BD Pharmingen). Data were acquired using a CyAn Analyser flow cytometer (Beckman Coulter, UK) and then analysed using Summit (Dako, UK) and FlowJo (Tree Star Inc, USA) software.

### Bone marrow transplantation

Ten-week-old male mice received a lethal dose of whole-body irradiation (2 × 5 Gy) followed by an i.v. injection of 5 × 10^6^ bone marrow cells from male *ApoE*^*−/−*^ and *Rgs1*^*−/−*^*ApoE*^*−/−*^ mice. As a control for the efficiency of the irradiation procedure and bone marrow transfer, bone marrow from CD45.1 antigen-expressing mice was transplanted to CD45.2 antigen-expressing mice and blood samples taken 4 weeks after engraftment and then antibody stained for the presence of the donor CD45.1 or recipient CD45.2 allele by flow cytometry. More than 95% blood cells expressed the donor CD45.1 antigen confirming successful reconstitution. Bone marrow transplantation resulted in a significant reduction in the incidence of aneurysms at 0.8 mg kg^−1^ per day Ang II. To achieve a similar degree of aneurysm to that of non-bone marrow-transplanted mice, we increased the dose of Ang II to 3 mg kg^−1^ per day after dosing studies.

### Inflammatory blood monocyte tracking

Inflammatory 7/4^hi^ monocytes were labelled *in vivo* by i.v. injection of 1 μm Fluoresbrite green fluorescent plain microspheres (Polysciences Inc., Germany) diluted 1:4 in saline, 18 h post i.v. injection of 200 μl clodronate liposomes (Clodronate Liposomes Org, Amsterdam)^23^,^24^. Labelling efficiency was confirmed by flow cytometry. Mice were then implanted with Ang II osmotic mini pumps (0.8 mg kg^−1^ per day). Flow cytometry was performed after 3 days (during the recruitment and clearance phase from the blood) and 5 days (during influx into aortic tissue) for quantification of bead-labelled cells in the aorta and blood.

### Immunofluorescence staining

Abdominal aortas from bead-injected mice were sectioned (10 μm) and blocked for 2 h in 5% goat serum, 1% BSA, 0.2% gelatin, 0.2% Triton-X-100 in PBS. Thereafter, the sections were incubated for 2 h with primary antibodies against Ly6C (BD Pharmingen) and 7/4 (AbD Serotec) followed by incubation with the secondary antibody Alexa Fluor 568 (Invitrogen; Supplementary Table 4). Sections were washed and mounted with mowial mounting medium with 4′,6-diamidino-2-phenylindole. Images were obtained with a Zeiss 510 MetaHead confocal fluorescence microscope.

### Statistical analysis

Between-group comparisons of normally distributed measurements were assessed by Student’s *t*-test. One-way analysis of variance was used to compare more than two data groups and Dunnett’s post-test was used to compare each group with a control (untreated) group. Two-way analysis of variance was used to compare multiple data groups affected by two independent variables, with a Bonferroni correction to compare groups with each other. Differences were considered statistically significant at *P*<0.05.

## Additional information

**Accession codes:** The microarray data has been deposited in the NCBI Gene Expression Omnibus under the accession code GSE65494.

**How to cite this article:** Patel, J. *et al*. RGS1 regulates myeloid cell accumulation in atherosclerosis and aortic aneurysm rupture through altered chemokine signalling. *Nat. Commun.* 6:6614 doi: 10.1038/ncomms7614 (2015).

## Supplementary Material

Supplementary Information Supplementary Figures 1-9 and Supplementary Tables 1-5
