# Supplementary material for: RGS1 regulates myeloid cell accumulation in atherosclerosis and aortic aneurysm rupture through altered chemokine signalling
Source: Nat Commun. 2015 Mar 18;6:6614. doi: 10.1038/ncomms7614 (PMC4374153; doi:10.1038/ncomms7614)
Supplement: Supplementary Information — Supplementary Figures 1-9 and Supplementary Tables 1-5 [file ncomms7614-s1.pdf]

Supplementary Figure 1

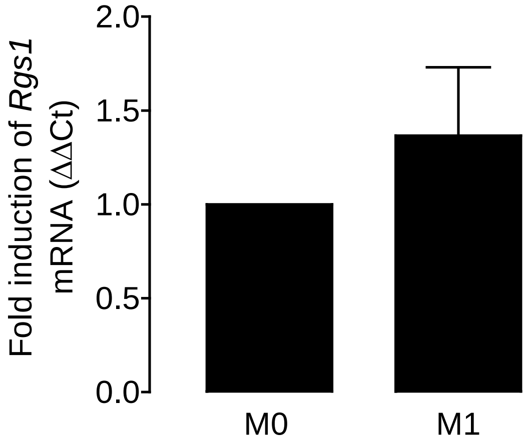

Supplementary Figure 1: *Rgs1* is expressed in human M1-polarised macrophages.
Human CD14+ monocytes were obtained from healthy human peripheral blood after monocyte isolation using magnetic separation (Miltenyi Biotec). Macrophages were obtained by culturing monocytes for 6 days then cultured for an additional 24 hours with or without 100 ng/ml LPS and 20 ng/ml IFN-γ (for M1 polarization). qRT-PCR analysis of *Rgs1* mRNA in M1 macrophages presented relative to mRNA in unstimulated cells (M0). (n=4).

Supplementary Figure 2

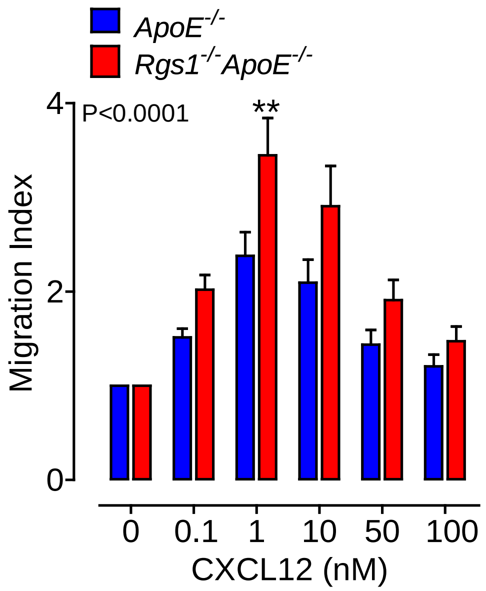

Supplementary Figure 2: RGS1 inhibits splenocyte migration to CXCL12. Migration of splenocytes from *ApoE*$^{-/-}$ and *Rgs1*$^{-/-}$ *ApoE*$^{-/-}$ mice through an 5 μm filter towards increasing concentrations of recombinant murine CXCL12 placed in the lower chamber of a Boyden chamber. Splenocytes (4 x 10^5 cells) were isolated after hypotonic lysis of red blood cells. Following a 3 hour incubation at 37°C, the number of cells that migrated into the lower chamber was determined by the CellTiter-Glo® luminescent cell viability assay (Promega, UK). Quantification of migration is presented relative to results of untreated cells, set as 1. RPMI media was used as a negative control. Graphs indicate migration index ± s.e.m for each treatment group in triplicate (n=10 per group). There was a significant difference between the two genotypes, P<0.0001 calculated by 2-way ANOVA. **, P<0.01 at 1 nM CXCL12, calculated by Bonferroni post-test.

Supplementary Figure 3

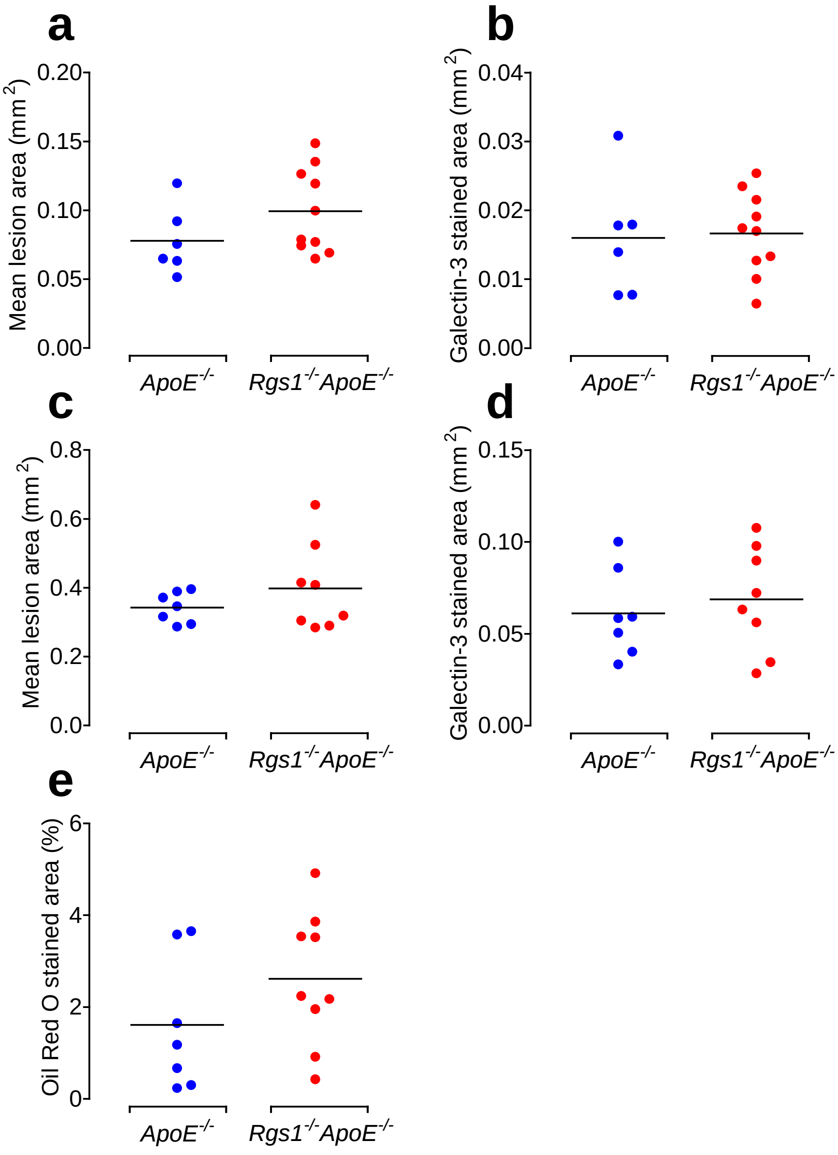

Supplementary Figure 3: *Rgs1* deficiency does not affect atherosclerosis and macrophage content in 16 week old and western-type diet fed *ApoE$^{-/-}$* mice.
(a) Atherosclerotic plaque in the aortic roots of 16-week old mice on a chow diet. (b) Galectin-3 positive macrophage content in the aortic roots of 16-week old mice on a chow diet. (c) Atherosclerotic plaque in the aortic roots of 16-week old mice on a western-type diet for 8 weeks. (d) Galectin-3 positive macrophage content in the aortic roots of 16-week old mice on a western-type diet for 8 weeks. (e) *En face* atherosclerotic plaque in the aortas of 16-week old mice on a western-type diet for 8 weeks. Each symbol represents an individual mouse (n=6-10). Values are expressed as mean ± s.e.m. There were no significant differences between groups.

Supplementary Figure 4

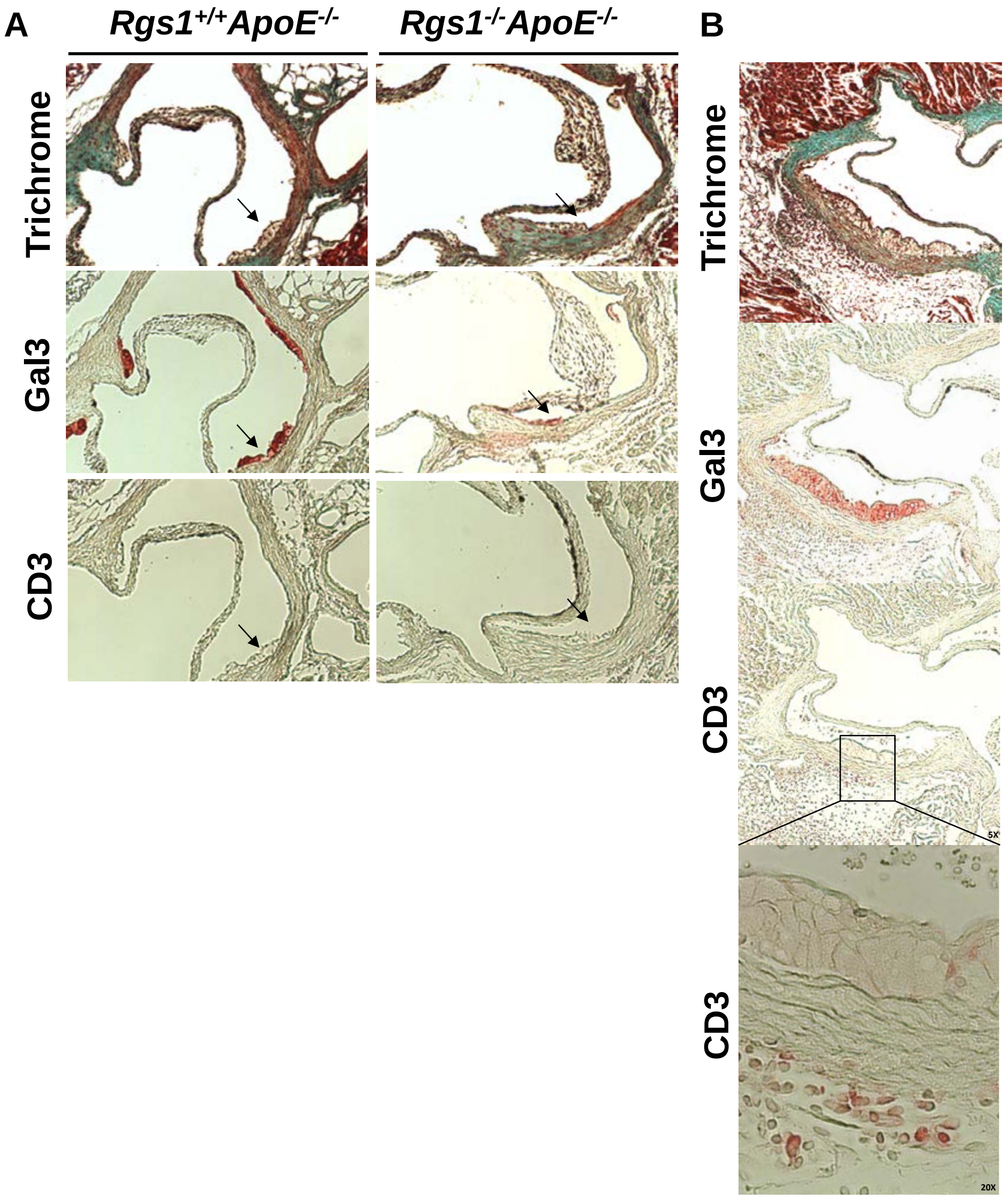

Supplementary Figure 4: Histological analysis of CD3 T-cell staining of aortic roots from *Rgs1*$^{+/+}$*ApoE*$^{-/-}$ and *Rgs1*$^{-/-}$*ApoE*$^{-/-}$ mice. (A) Representative images of aortic root sections from 9 week old *Rgs1*$^{+/+}$*ApoE*$^{-/-}$ and *Rgs1*$^{-/-}$*ApoE*$^{-/-}$ mice on a chow diet that were stained for Massons Trichrome for lesion analysis, Galectin-3 for macrophage content and CD3 for T-cells. No significant CD3 staining was observed in aortic roots at 9 weeks. Rgs1 deficient mice had smaller, or no lesions compared to *ApoE*$^{-/-}$ mice at this stage. (B) Confirmation of positive CD3 staining in the adventitia behind aortic root lesions of 16 week old mice on a chow diet. Representative images of aortic root sections from an *ApoE*$^{-/-}$ mouse on a chow diet for 16 weeks, that were stained for Massons Trichrome for lesion analysis, Galectin-3 for macrophage content and CD3 for T-cells.

Supplementary Figure 5

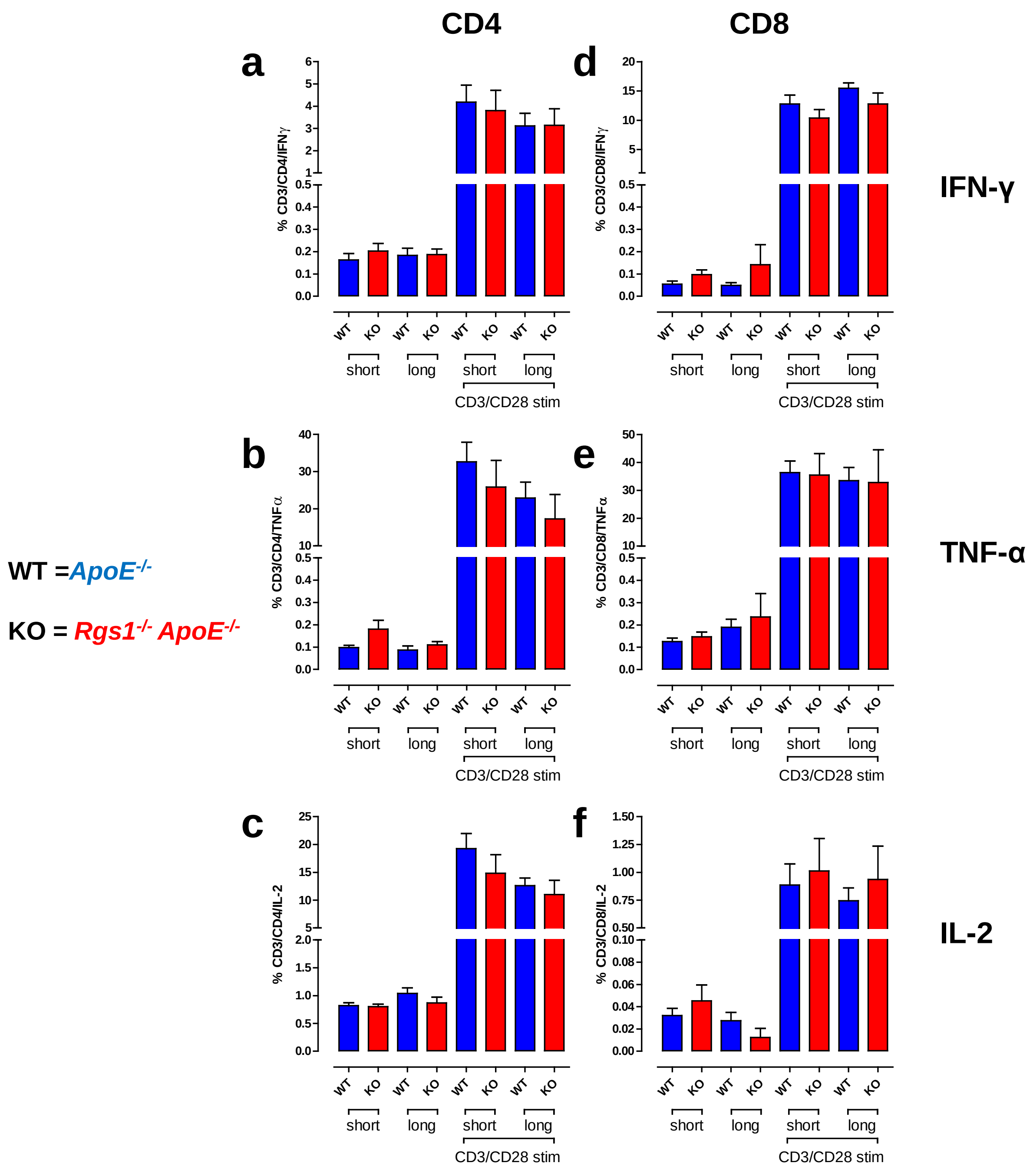

Supplementary Figure 5: *Rgs1* deficiency does not affect a Th1 cell phenotype. Splenocytes (3 x 10^6 cells) were isolated after hypotonic lysis of red blood cells and incubated for 4 hours (short) or overnight (long) with anti-CD3/CD28 co-stimulation at 37°C. Cells were centrifuged, washed and then stained with live/dead fixable stain (Life Technologies) then incubated in cell surface specific antibodies and Fc block. For staining of intracellular markers, cells were washed, permeabilised by incubation with Cytofix/Cytoperm then washed in PermWash (BD Biosciences). The percentage of Th1 cytokines; IFN-γ, TNF-α and IL-2 expressing (a-c) CD4 and (d-f) CD8 cells were quantified by flow cytometry (n=4 per group).Values are expressed as mean ± s.e.m. There were no significant differences between the two genotypes.

Supplementary Figure 6

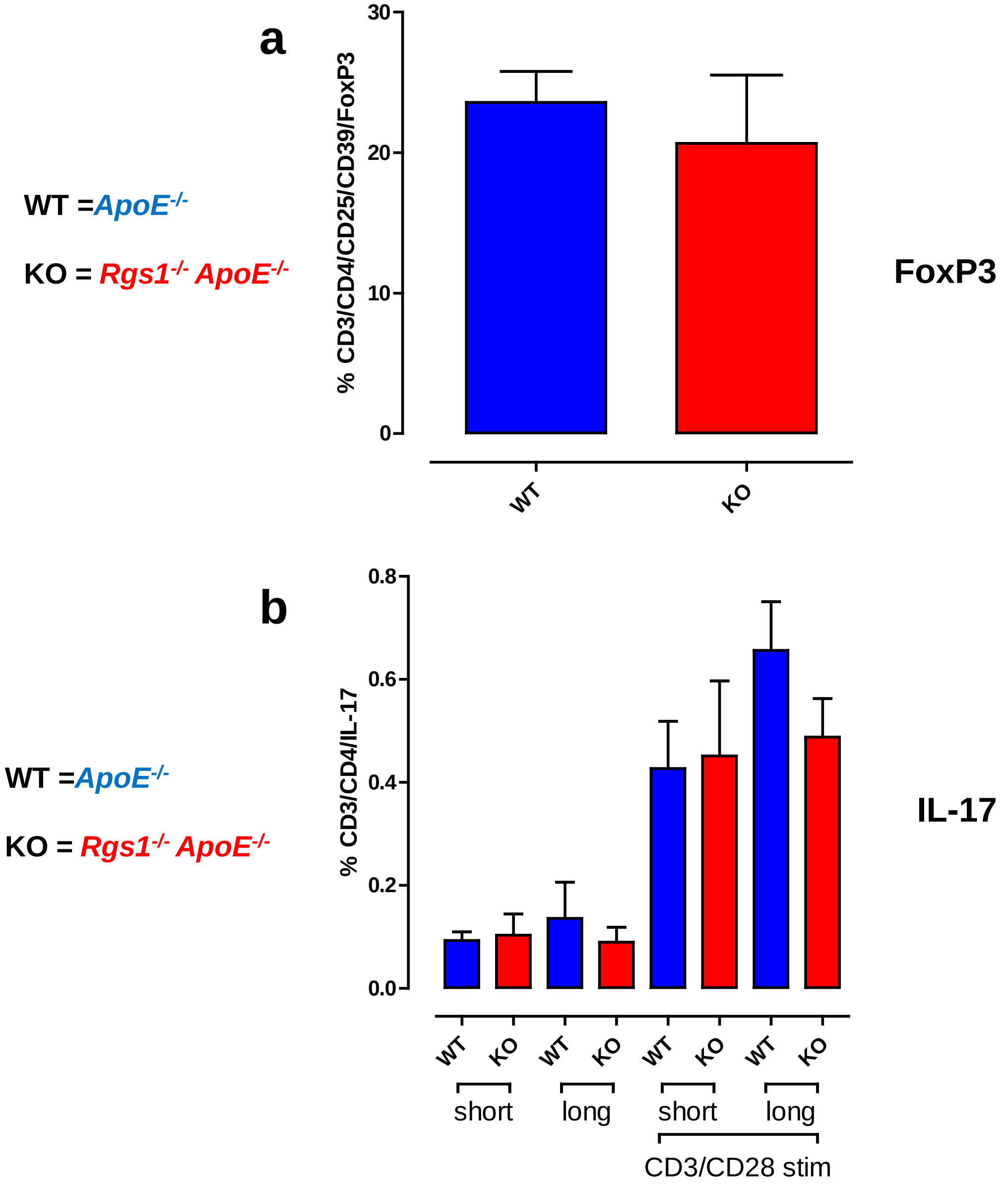

Supplementary Figure 6: *Rgs1* deficiency does not affect a Treg cell and Th17 cell phenotype.

Splenocytes (3 x 10^6 cells) were isolated after hypotonic lysis of red blood cells and (a) the percentage of FoxP3 expressing CD39+CD25+CD4+CD3+ cells were quantified by flow cytometry (n=4 per group) (b) incubated for 4 hours (short) or overnight (long) with anti-CD3/CD28 stimulation at 37°C. Cells were centrifuged, washed and then stained with live/dead fixable stain (Life Technologies) then incubated in cell surface specific antibodies and Fc block. For staining of intracellular markers, cells were washed, permeabilised by incubation with Cytofix/Cytoperm then washed in PermWash (BD Biosciences). The percentage of IL-17 expressing CD4+CD3+ cells were quantified by flow cytometry (n=4 per group).Values are expressed as mean ± s.e.m. There were no significant differences between the two genotypes.

Supplementary Figure 7

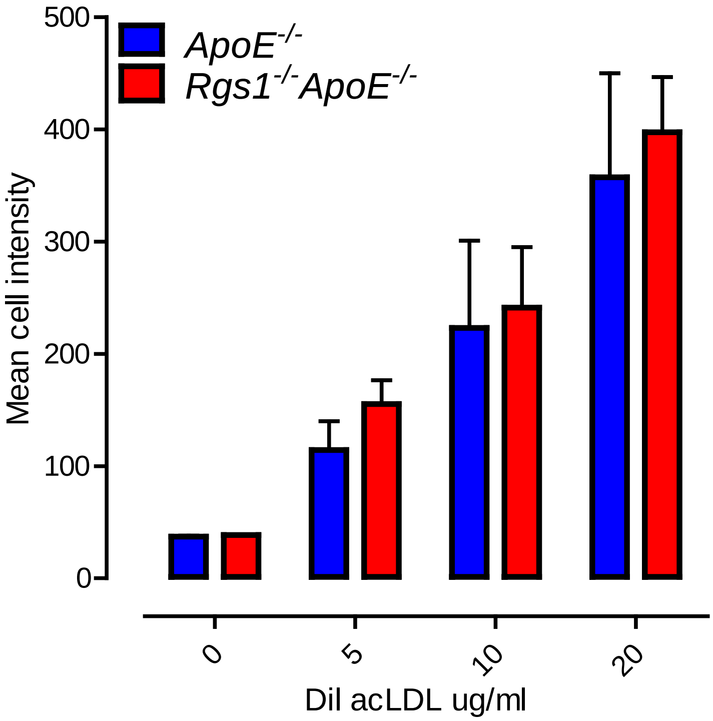

Supplementary Figure 7: **RGS1 deficiency does not affect foam cell formation.** Bone marrow derived macrophages (1 x 10^5 cells) from *ApoE*$^{-/-}$ and *Rgs1*$^{-/-}$ *ApoE*$^{-/-}$ mice were cultured for 7 days prior to treatment with 5, 10 and 20 ug/ml Dil-acLDL for 24 hours. Quantification of LDL uptake was measured by fluorescent intensity per cell over 5 fields per well using the Operetta High Content Imaging System. Graphs indicate mean cell intensity ± s.e.m for each dose in quadruplicate (n=3 per group, representative of two separate experiments). There were no significant differences between groups.

Supplementary Figure 8

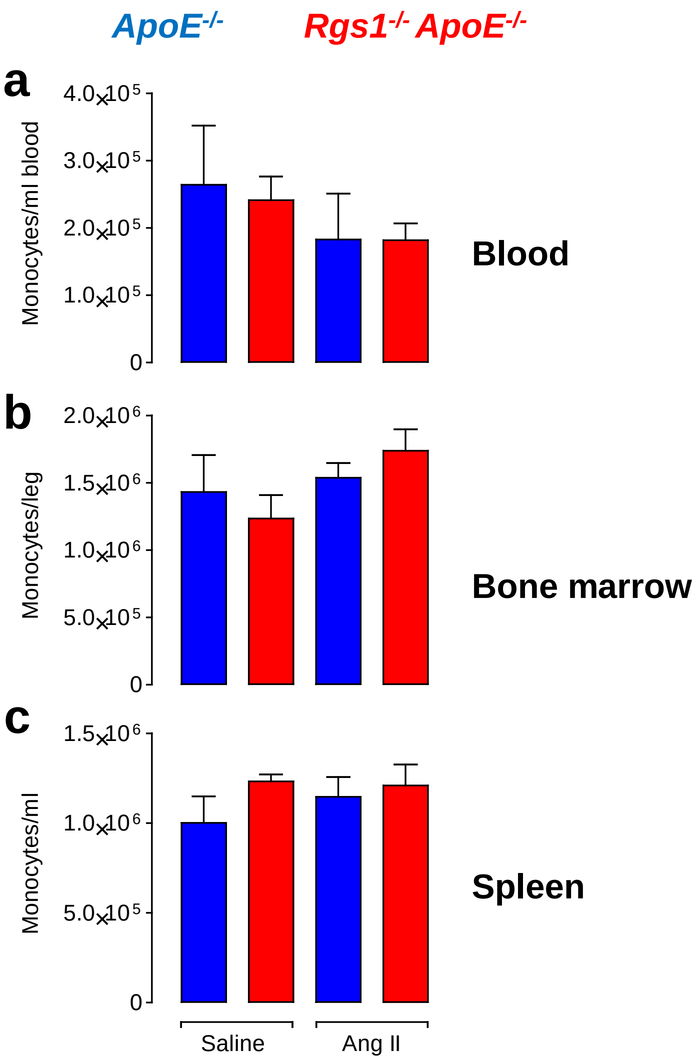

Supplementary Figure 8: *Rgs1* deficiency does not affect monocyte numbers in the blood, bone marrow or spleen of *ApoE*$^{-/-}$ mice after Ang II infusion.
Leukocyte counts from (a) blood, (b) bone marrow and (c) spleen in 9 week old *ApoE*$^{-/-}$ and *Rgs1*$^{-/-}$ *ApoE*$^{-/-}$ mice after 14 days of Ang II infusion at 0.8 mg/kg/day. Monocytes from indicated tissues were quantified by flow cytometry and identified as CD11b^{+}Ly6G^{-} 7/4^{hi} cells. There was no overall significant effect of *Rgs1* deficiency on monocyte numbers in the blood, bone marrow or spleen (n=2-3 for saline and 7-13 for Ang II per group). Values are expressed as mean ± s.e.m.

Supplementary Figure 9

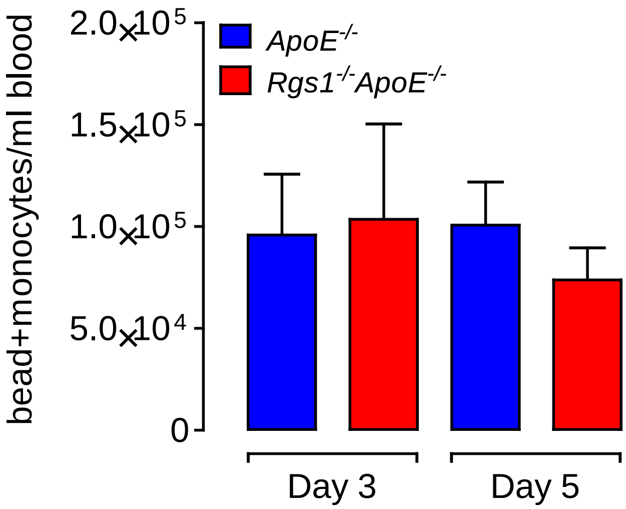

Supplementary Figure 9: *Rgs1* deficiency does not affect inflammatory monocyte numbers in the blood after Ang II infusion.

ApoE$^{-/-}$ and *Rgs1*$^{-/-}$ *ApoE*$^{-/-}$ mice received Ang II infusion at 0.8 mg/kg/day for 3 and 5 days following fluorescent bead labeling of circulating inflammatory monocytes. The numbers of bead labeled circulating 7/4^{hi} monocytes at day 3 and day 5 were quantified by flow cytometry (n=5-7 per group). Monocytes were identified as CD45^{+}CD11b^{+}7/4^{hi} cells. Values are expressed as mean ± s.e.m.

Supplementary Table 1

Genbank	Name	Change in expression of gene in thoracic aorta between 8 and 16 weeks	
		Fold Change	P value
NM_009263	Spp1	24.88	0.000133
NM_008491	Lcn2	3.52	0.000292
NM_011333	Ccl2	3.53	0.000392
NM_010444	Nr4a1	0.45	0.00043
NM_011593	Timp1	2.38	0.0011
NM_172750	Adprhl1	0.49	0.00139
NM_153122	Oplah	0.68	0.00206
NM_008764	Tnfrsf11b	2.11	0.00213
NM_021455	Mlxipl	0.54	0.00512
NM_015811	Rgs1	1.74	0.00512
NM_007734	Col4a3	0.55	0.00675
NM_025999	Rnf141	0.69	0.00749
NM_011251	Rbm6	1.24	0.00749
NM_023124	H2-Q8	0.39	0.0075
NM_024221	Pdhb	0.57	0.00908
NM_021400	Prg4	5.77	0.00908
NM_018884	Pdzrn3	1.39	0.0113
NM_010391	H2-Q10	0.30	0.0122
NM_010391	H2-Q10	0.34	0.0122
Poln	DNA polymerase N	0.40	0.0122
NM_022322	Tnmd	0.42	0.0122
	Tmem120b (LOC330189)	0.53	0.0122
NM_024446	Nudt7	0.58	0.0122
	SIMILAR TO GALE	0.72	0.0122
	Adipor2	0.72	0.0122
NM_009427	Tob1	0.76	0.0122
NM_011570	Tes	1.47	0.0122
NM_011511	Abcc9	1.53	0.0122
	CSMD2	2.10	0.0122
NM_021281	Ctss	4.03	0.0122
NM_028069	Mucdhl	0.71	0.0137
NM_013473	Anxa8	2.43	0.0154
NM_008409	Itm2a	0.49	0.0166
NM_153508	Clstn3	0.50	0.0169
NM_139292	Reep6	0.62	0.0185
NM_030210	Aacs	0.62	0.0185
NM_133891	Slc44a1	0.72	0.0185
NM_144784	Acat1	0.78	0.0185
NM_007920	Elf1	1.28	0.0185
NM_009423	Traf4	0.74	0.0191
	ECMR-III	1.60	0.0191
NM_011693	Vcam1	2.50	0.0191
NM_025297	Mecr	0.64	0.0195
NM_011620	Tnnt3	11.57	0.0196
NM_008284	Hras1	0.49	0.0197
NM_013935	Ptpla	0.69	0.0197
	FLAVOHEMOPROTEIN B5/B5R.	1.32	0.0201
NM_022019	Dusp10	0.69	0.0203
NM_009760	Bnip3	0.77	0.021
NM_053200	ESTERASE- 22	0.49	0.0214
NM_007751	Cox8b	0.39	0.022
	P56-LCK	0.41	0.022
NM_019811	Acss2	0.54	0.022
	OROSOMUCOID 3	0.57	0.022
	SIMILAR TO GLUTAMIC-PYRUVATE TRANSAMINASE	0.57	0.022
NM_009948	Cpt1b	0.59	0.022
NM_009171	Shmt1	0.60	0.022
NM_024450	Scd3	0.65	0.022
NM_025369	Mrps36	0.78	0.022
NM_023813	Camk2d	1.24	0.022
NM_031256	Plekha3	1.32	0.022
NM_030690	Rai14	1.33	0.022
NM_026835	Ms4a6d	2.27	0.022
NM_011693	Vcam1	2.33	0.022
NM_009252	Serpina3n	2.39	0.022
NM_009196	Slc16a1	0.60	0.0221
NM_016895	Ak2	0.72	0.0221
NM_026769	Drd1ip	0.47	0.0223
NM_019760	Serinc1	1.26	0.0223
NM_019391	Lsp1	1.53	0.0223
	Crlf-1 Acc:Q9D2R5	2.03	0.0223
NM_031180	Klb	0.50	0.0232
NM_007654	Cd72	2.01	0.0232
NM_016754	Mylpf	6.08	0.0232
NM_011315	Saa3	6.51	0.0232
	SIMILAR TO OUT AT FIRST PROTEIN (HYPOTHETICAL PROTEIN)	0.70	0.0246
NM_007639	Cd1d1	0.51	0.0248
NM_173007	Tspan12	0.55	0.0259
NM_009982	Ctsc	1.31	0.0259
NM_011890	Sgcb	1.25	0.0275
NM_020025	B3galt2	0.50	0.0276
NM_008452	Klf2	0.63	0.0278
NM_017398	Diap2	1.34	0.0287
NM_010819	Clec4d	2.76	0.0287
NM_144961	MYOSIN HEAVY CHAIN 2A	25.39	0.0287
	TCR BETA-2 CHAIN C REGION	0.37	0.0294
	NGAL Acc:P11672]	2.58	0.03
NM_145570	BC014699	0.66	0.0307
NM_013654	Ccl7	2.27	0.0307
NM_030888	C1qtnf3	0.49	0.0309
NM_153779	Amid	0.61	0.0309

NM_009204	Slc2a4	0.62	0.0309
NM_026981	Dtwd1	1.28	0.0309
NM_173413	Rab8b	1.37	0.0311
NM_130450	Elovl6	0.44	0.0314
NM_029844	Mrap	0.54	0.0314
NM_026443	1700020C11Rik	0.64	0.0335
NM_011353	Serf1	0.70	0.0335
NM_138597	Atp5o	0.81	0.0335
NM_025375	Wbscr22	1.31	0.0335
NM_176919	Ppm1h	1.37	0.0354
	TRANSCRIPTIONAL REPRESSOR PAR-4-LIKE PROTEIN PAWR (FRAGMENT).	1.57	0.0354
NM_011331	Ccl12	3.02	0.0354
NM_026071	Slc25a19	0.74	0.0373
NM_020519	Slurp1	3.03	0.038
NM_019584	Becn1	1.27	0.0381
NM_008604	Mme	0.60	0.0383
NM_029620	Pcolce2	0.70	0.0383
NM_028017	NAPG	1.32	0.0383
NM_022992	Arl6ip5	1.31	0.039
NM_009669	Amy2	0.62	0.0391
NM_013532	Lilrb4	3.09	0.0392
NM_007544	Bid	1.30	0.04
NM_032002	Nrg4	0.46	0.0409
NM_019744	Ncoa4	1.41	0.0411
NM_009779	C3ar1	2.16	0.0416
NM_008797	Pcx	0.50	0.0417
NM_017379	Tuba8	0.56	0.0417
NM_146125	Itpka	0.59	0.042
NM_177034	Apba1	1.28	0.042
NM_008845	Pip5k2a	1.40	0.042
NM_025348	Ndufa3	0.77	0.0429
NM_020582	Atp5j2	0.70	0.0434
NM_026840	Pdgfrl	0.65	0.0438
NM_022332	St7	0.86	0.0438
NM_025843	Ndufb7	0.92	0.0438
NM_023665	D4Wsu53e	1.60	0.0438
NM_007590	Calm3	1.20	0.044
NM_031254	Trem2	3.15	0.044
NM_009115	S100b	0.64	0.0459
NM_153799	Lsm16	0.80	0.0459
NM_010781	Mcpt6	0.72	0.0463
NM_009367	Tgfb2	1.42	0.0463
NM_053110	Gpnmb	4.33	0.0463
NM_011281	Rorc	0.52	0.0465
NM_024208	Echdc3	0.73	0.0465
NM_138601	D10Jhu81e	0.74	0.0465
NM_019640	Pitpnb	1.33	0.0465
NM_145575	Cald1	1.92	0.0465
NM_007703	Elovl3	0.39	0.048
NM_145122	Pex16	0.71	0.0495
	CYSTEINE-RICH REPEAT-CONTAINING PROTEIN CRIM1 PRECURSOR (FRAGMENT)	1.54	0.0495
NM_011176	St14	1.30	0.0496
NM_144539	Slamf7	2.83	0.0496
	XAP-5-LIKE PROTEIN	0.41	0.0499
NM_011585	Tia1	1.37	0.0499
NM_025427	1190002H23Rik	0.56	0.05
NM_134046	2810429O05Rik	0.68	0.05
	C8g	0.75	0.05
NM_025930	2600011C06Rik	1.24	0.05

Supplementary Table 1: Regulated genes in thoracic aortas of 16 week old *ApoE*$^{-/-}$ mice fed a western type diet compared to 8 week old *ApoE*$^{-/-}$ mice.

Gene expression was measured using a whole mouse gene array. Statistical analysis was performed using GeneSpring software with correction for multiple comparisons. 150 genes showed significantly different expression in the 16 week old mice compared to 8 week old *ApoE*$^{-/-}$ mice, P value<0.05. 16 of these genes including RGS1 were differentially expressed with a P value<0.01.

Supplementary Table 2

	ApoE$^{-/-}$	*Rgs1*$^{-/-}$*ApoE*$^{-/-}$
**Blood (cells x10^4/ml)**		
Monocytes	8.09 ±3.65	5.77 ±1.71
Neutrophils	13.37 ±7.10	14.13 ±2.48
CD3 T cells	41.10 ±14.61	57.48 ±18.70
CD4 T cells	22.86 ±9.37	30.13 ±6.91
CD8 T cells	12.11 ±5.94	17.5 ±6.23
B cells	65.85 ±18.37	66.45 ±18.35
**Bone marrow (cells x10^6/leg)**		
Monocytes	0.38 ±0.19	0.53 ±0.28
Neutrophils	2.65 ±1.15	2.89 ±1.74
CD3 T cells	0.18 ±0.11	0.17 ±0.08
CD4 T cells	0.06 ±0.04	0.05 ±0.02
CD8 T cells	0.06 ±0.01	0.07 ±0.01
B cells	0.86 ±0.08	0.93 ±0.68
**Spleen (cells x 10^5/ml)**		
Monocytes	2.35 ±1.20	1.19 ±0.56 *
Neutrophils	4.80 ±1.81	3.31 ±1.85
CD3 T cells	22.56 ±5.63	23.30 ±5.27
CD4 T cells	16.98 ±4.35	16.26 ±4.27
CD8 T cells	4.22 ±1.34	5.60 ±2.68
B cells	27.58 ±12.11	21.88 ±11.08

Supplementary Table 2: *Rgs1* deficiency does not affect homeostatic cell numbers in the blood or bone marrow of *ApoE*$^{-/-}$ mice.

Leukocyte counts from blood, bone marrow and spleen in 9 week old *ApoE*$^{-/-}$ and *Rgs1*$^{-/-}$*ApoE*$^{-/-}$ mice on a chow diet were quantified by flow cytometry. Monocytes were identified as CD11b^{+}Ly6G^{-}7/4^{hi} cells and neutrophils as CD11b^{+}Ly6G^{+}7/4^{hi} cells. T cells were identified as CD3^{+} cells, then CD4 or CD8 T cells and B cells were identified as B220^{+} cells. There was no overall significant effect of *Rgs1* deficiency on cell numbers in the blood or bone marrow but a difference in monocytes in the spleen (n=5-8 per group). Values are expressed as mean ± s.d. *P <0.05 calculated using the Students t-test.

Supplementary Table 3

Gene	Reference Sequence Accession no.	ABI product number
Mouse Rgs1	NM_015811.2	Mm01286234_g1
Mouse Cd68	NM_009853.1	Mm00839636_g1
Human Rgs1	NM_002922.3	Hs00175260_m1
Human GAPDH	NM_002046.3	Hs02758991_g1
Mouse β-actin	N/A	4352341E

Supplementary Table 3: Primers for qRT-PCR
All assays used in qRT-PCR are listed with accession numbers and product number. All TaqMan® probes were designed and optimized by Applied Biosystems (ABI; Warrington, UK). All are commercial products; sequences not available.

Supplementary Table 4

Name	Concentration	Product number	Supplier
Goat polyclonal anti-mouse Galectin-3 antibody	1:500	AF1197	R&D Systems
Rabbit polyclonal anti-human CD-3 antibody	1:100	A0452	Dako
Mouse Monoclonal α-Smooth muscle actin clone 1A4	1:50	A-5691	Sigma-Aldrich
Biotinylated anti-goat IgG	1:200	BA-5000	Vector Labs
Biotinylated anti-rabbit IgG	1:200	BA-1000	Vector Labs
Alexa Fluor® 568 Goat Anti-Rat IgG	1:500	A-11077	Life Technologies

Supplementary Table 4: Antibodies used in immunohistochemical staining

Supplementary Table 5

Target	Conjugate	Clone	Working Concentration	Product number	Supplier
CD16/CD32	purified	2.4G2	5 µg/ml	553142	BD Biosciences
CD11b	PerCP Cy5.5	M1/70	1 µg/ml	550993	BD Biosciences
Ly6B.2 (7/4)	PE	7/4	2 µg/ml	MCA771PE	AbD Serotec
Ly6G	FITC	1A8	2.5 µg/ml	551460	BD Biosciences
B220	PE	RA3-6B2	1 µg/ml	553089	BD Biosciences
B220	PE-Texas Red	RA3-6B2	0.7 µg/ml	551489	BD Biosciences
CD3	Pacific Blue	500A2	1 µg/ml	558214	BD Biosciences
CD3	Per CP Cy5.5	145-2c11	1 µg/ml	45-0031-82	eBioscience
CD3	Alexa Fluor 700	17A2	1.25 µg/ml	100216	Biolegend
CD4	Alexa Fluor 647	RM4-5	1 µg/ml	557681	BD Biosciences
CD4	efluor 650 NC	GK1.5	2 µg/ml	95-0041-42	eBioscience
CD8	FITC	53-6.7	1 µg/ml	553030	BD Biosciences
CD8	APC-ef780	53-6.7	2 µg/ml	47-0081-82	eBioscience
CD45	PE-Cy7	30-F11	1 µg/ml	552848	BD Biosciences
CD14	PE	Sa2-8	0.25 µg/ml	12-0141-81	eBioscience
CCR2	APC	475301	0.2 µg/ml	FAB5538A	R&D Systems
CCR5	PE	HM-CCR5	2 µg/ml	107005	Biolegend
IFN-γ	Brilliant violet 421	XMG1.2	0.25 µg/ml	505829	Biolegend
TNF-α	FITC	MP6-XT22	2.5 µg/ml	53-7321-82	eBioscience
IL-2	Pe-Cy7	JES6-5H4	1 µg/ml	25-7021-82	eBioscience
IL-17a	Per CP Cy5.5	eBio17B7	3 µg/ml	45-7177-82	eBioscience
CD25	APC	PC61.5	0.5 µg/ml	17-0251-82	eBioscience
CD39	Pe-Cy7	24DMS1	2 µg/ml	25-0391-82	eBioscience
FoxP3	PE	FJK-16S	2 µg/ml	12-5773-82	eBioscience

Supplementary Table 5: Antibodies used for flow cytometry
